# A conserved role for Syntaxin-1 in pre- and post-commissural midline axonal guidance in fly, chick, and mouse

**DOI:** 10.1371/journal.pgen.1007432

**Published:** 2018-06-18

**Authors:** Oriol Ros, Pablo José Barrecheguren, Tiziana Cotrufo, Martina Schaettin, Cristina Roselló-Busquets, Alba Vílchez-Acosta, Marc Hernaiz-Llorens, Ramón Martínez-Marmol, Fausto Ulloa, Esther T. Stoeckli, Sofia J. Araújo, Eduardo Soriano

**Affiliations:** 1 Department of Cell Biology, Physiology and Immunology, Faculty of Biology and Institute of Neurosciences, University of Barcelona, Barcelona, Spain; 2 Centro de Investigación Biomédica en Red sobre Enfermedades Neurodegenerativas (CIBERNED), ISCIII, Madrid, Spain; 3 Institut de Recerca Biomedica de Barcelona (IRB Barcelona), Parc Cientific de Barcelona, Barcelona, Spain; 4 Institute of Molecular Life Sciences and Neuroscience Center Zurich, University of Zurich, Zurich, Switzerland; 5 Institut de Biologia Molecular de Barcelona (IBMB-CSIC), Parc Cientific de Barcelona, Barcelona, Spain; 6 Department of Genetics, Microbiology and Statistics, Faculty of Biology, University of Barcelona, Barcelona, Spain; 7 Vall d´Hebron Institute of Research (VHIR), Barcelona, Spain; 8 Institució Catalana de Recerca i Estudis Avançats (ICREA), Barcelona, Spain; Fred Hutchinson Cancer Research Center, UNITED STATES

## Abstract

Axonal growth and guidance rely on correct growth cone responses to guidance cues. Unlike the signaling cascades that link axonal growth to cytoskeletal dynamics, little is known about the crosstalk mechanisms between guidance and membrane dynamics and turnover. Recent studies indicate that whereas axonal attraction requires exocytosis, chemorepulsion relies on endocytosis. Indeed, our own studies have shown that Netrin-1/Deleted in Colorectal Cancer (DCC) signaling triggers exocytosis through the SNARE Syntaxin-1 (STX1). However, limited *in vivo* evidence is available about the role of SNARE proteins in axonal guidance. To address this issue, here we systematically deleted SNARE genes in three species. We show that loss-of-function of STX1 results in pre- and post-commissural axonal guidance defects in the midline of fly, chick, and mouse embryos. Inactivation of VAMP2, Ti-VAMP, and SNAP25 led to additional abnormalities in axonal guidance. We also confirmed that STX1 loss-of-function results in reduced sensitivity of commissural axons to Slit-2 and Netrin-1. Finally, genetic interaction studies in *Drosophila* show that STX1 interacts with both the Netrin-1/DCC and Robo/Slit pathways. Our data provide evidence of an evolutionarily conserved role of STX1 and SNARE proteins in midline axonal guidance *in vivo*, by regulating both pre- and post-commissural guidance mechanisms.

## Author summary

Syntaxin-1 is a core factor in tethering synaptic vesicles and mediating their fusion to the cell membrane at the synapse. Thus, Syntaxin-1 mediates neurotransmission in the adult nervous system. Here we show that this protein is also involved in axonal guidance in the CNS of vertebrates and invertebrates during the development of the nervous system: our systematic analysis of the phenotypes in the nervous system midline of fly, chick, and mouse embryos mutant for Syntaxin-1 unveils an evolutionarily conserved role for this protein in midline axonal guidance. Further, we also dissect the contribution of other proteins regulating neuronal exocytosis in axonal development. We propose that the coupling of the guidance molecule machinery to proteins that regulate exocytosis is a general mechanism linking chemotropism to axonal growth.

## Introduction

Axonal growth and guidance are responsible for the correct formation of neural circuits. These processes rely on the tightly regulated response of the growth cone to both diffusible and membrane-bound guidance cues. In response to such cues, several intracellular signaling cascades are activated within the growth cone, leading to directional growth. For instance, the chemoattractant Netrin-1 binds to the receptor Deleted in Colorectal Cancer (DCC) at growth cones, activating several kinases and small GTPases, cyclic nucleotides, and calcium cascades, as well as cytoskeletal rearrangements [1–8].

In contrast, few reports have addressed the cross-talk mechanisms between axonal guidance and membrane dynamics and turnover, other than the fact that growth cones are filled by vesicles and express most SNARE (Soluble NSF Attachment Protein REceptor) and exocyst proteins [9–13]. A growing number of reports using *in vitro* approaches indicate that axon guidance mechanisms require the participation of SNARE-mediated exocytosis for chemoattraction and endocytosis for repulsion [14–18]. Thus, it has been demonstrated that the vSNARE (vesicular SNARE) VAMP2 is required for L1-mediated chemoattraction [19] and for Sema3A-induced chemorepulsion [17], that the vSNARE Ti-VAMP and the tSNARE (target SNARE) SNAP25 are necessary for neurite outgrowth [20–22], and that Syntaxin-1 (STX1) is required for Netrin-1-mediated attraction of axons and migrating neurons [15,16].

However, the participation of these proteins in neural circuit formation *in vivo* is still controversial. For instance, mice deficient for the SNAP25 and VAMP2 proteins show virtually no neural circuitry defects but do present a severe alteration of evoked synaptic activity [23–25]. Ti-VAMP-deficient mice display behavioral defects but no alterations in gross brain morphology [26]. STX1A knock-out (KO) mice show only mild cognitive defects and a normal brain structure [27] and axonal defects have not been described in STX1B KO [28]. In a previous study we showed that STX1A is required for the navigation of dorsal commissural in the chick spinal cord [16]. Syntaxin-1 loss-of-function in *Drosophila* and chick embryos results in motor axonal defects [29].

*Drosophila melanogaster* displays neural expression of a synaptobrevin (VAMP) gene, namely *n-synaptobrevin* (*n-syb*) [30] and a SNAP25 homolog, *Snap25* [31]. Mutations in these components of the core SNARE complex give rise to neurotransmitter release phenotypes [32]. A single *D*. *melanogaster* STX1 homolog, *Syntaxin1A* (*Syx1A)*, has been identified that shows strong homology to rodent STX1A [33–35]. Mutations in this gene are homozygous lethal, with severe alleles resulting in early embryonic death. In *D*. *melanogaster*, loss of *Syx1A* abolishes synaptic transmission [33,36], and other secretion phenotypes, such as soft cuticle and undigested yolk, have also been reported [33]. In addition, *Syx1A* is involved in cell membrane formation during cellularization [37] and in the condensation of the embryonic CNS [33]. In addition, *Syx1A* has been reported to affect the properties of neuronal membranes and influence membrane dynamics throughout development [38]. However, in *D*. *melanogaster*, in contrast to vertebrates, *Syx1A* has yet to be directly implicated in axonal guidance.

Here we systematically inactivated SNARE genes in three species, *Drosophila melanogaster*, chick, and mouse, to determine the role of SNARE proteins in CNS midline axonal guidance *in vivo*. We show the involvement of this protein complex, in particular STX1, in *D*. *melanogaster* and chicken axonal guidance, reporting aberrant phenotypes. Furthermore, we provide the first description of abnormal midline axonal defects in mouse embryos double mutant for STX1A and STX1B. Our results point to an evolutionarily conserved mechanism of SNARE complex proteins in midline axonal guidance.

## Results

### Loss of function of *Syx1A* causes specific axon guidance defects in *D*. *melanogaster*

To determine whether Syx1A affects axon guidance at the midline of *D*. *melanogaster* embryos, we started by analyzing the CNS phenotypes of *Syx1A* mutant embryos. For this purpose, we used zygotic mutants, since *Syx1A* has a maternal contribution and its function is required for proper cellularization [37].

**Fig 1:**
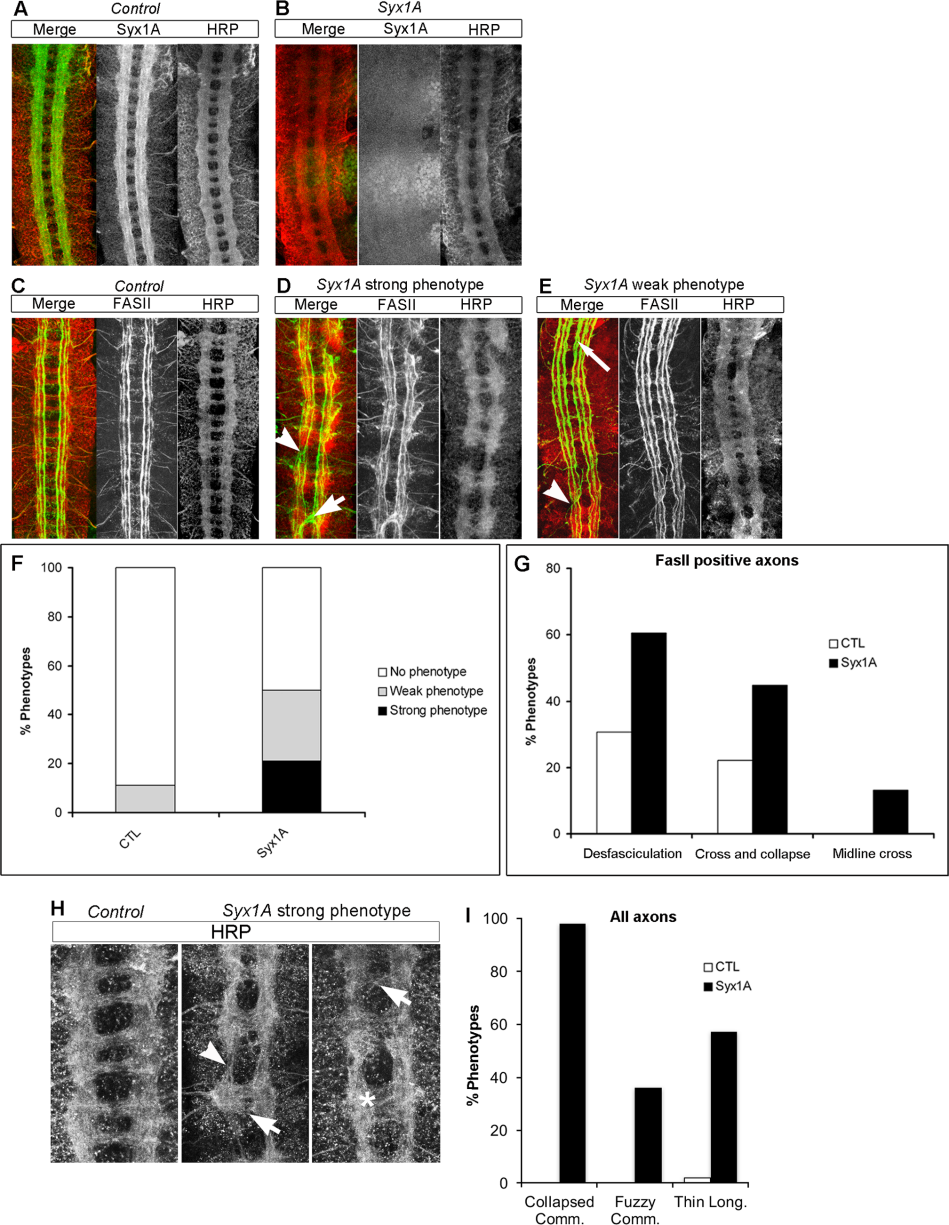
Axonal guidance defects found in the VNC of *Syx1A* mutant embryos. (A, B) Stage 16 embryos were stained with anti-Syx1A to detect Syntaxin 1 localization and with HRP to visualize the axon scaffold. A, Syx1A is detected in all axonal tracts, both commissural and longitudinal. B, Syx1A is not detected at these stages in *Syx1A*^*Δ229*^ mutant embryos. (C-E) Stage 16 embryos were stained with the anti-FasII mAb 1D4 to mark all FasII-positive axons and with HRP to visualize the axon scaffold. C, wt embryo showing the FasII-positive longitudinal connectives and the anterior and posterior commissures. D, *Syx1A*^*Δ229*^ mutant embryo representative of the strongest CNS phenotypes encountered; many commissures and longitudinal connectives are collapsed (arrowhead) and midline crosses (arrow) are observed. E, *Syx1A*^*Δ229*^ mutant embryo representative of the milder CNS phenotypes encountered; commissures are generally unaffected and defects are detected only in the longitudinal connectives, such as defasciculation (long arrow) and collapse (arrowhead). Anterior is up in all panels. (F) Quantification of the number of axon guidance defects encountered in *Syx1A*^*Δ229*^ mutants in comparison with the background defects in the wt. (G) Quantification of the diverse axonal guidance defects encountered in FasII positive axons in *Syx1A*^*Δ229*^ mutants in comparison with the background defects in the wt. (H) Higher magnification of *Syx1A*^*Δ229*^ mutant embryos representative of the wt and the strongest CNS phenotypes encountered by staining all axons with HRP; most commissures (98%) are thinner than in the wt; many segments (36%) show “fuzzy” commissures with a clear lack of separation between anterior commissure (AC) and posterior commissure (PC) (arrows and asterisk); longitudinal connectives are thinner between segments (arrowheads; 57% of cases) n = 120 segments; anterior is up. (I) Quantification of the axonal guidance defects encountered in all axons of *Syx1A*^*Δ229*^ mutants in comparison with the background defects in the wt when all axons are stained using anti-HRP antibody.

In *D*. *melanogaster*, CNS axons of wild-type (wt) embryos are found in a stereotypic ladder-like arrangement. Within each neuromere, two commissures link the two halves of the nervous system. Individual neuromeres are connected by axons running in discrete fascicles in the lateral connectives. An antiserum that specifically recognizes *D*. *melanogaster* Syx1A revealed that, like Frazzled (Fra, the *D*. *melanogaster* DCC homolog) [39], *Syx1A* is expressed in developing axons in the embryo from stage 13. At later stages, this isoform is expressed at high levels on commissural and longitudinal axons in the developing CNS (Fig 1A). Embryos of the null *Syx1A*^*Δ229*^ genotype [33] displayed no detectable Syx1A protein in the ventral nerve cord (VNC) when stained with the same antibody (Fig 1B).

To analyze axonal midline phenotypes, we stained embryos with HRP, which marks all axons in the CNS, and anti-Fasciclin II antibodies (anti-FasII), which label lateral fascicles (Fig 1C). At stage 15 or later, FasII identifies three major axonal tracts, which are visible as parallel straight lines: the medial (FasII-m or 1^st^), intermediate (FasII-i or 2^nd^) and lateral (FasII-l or 3^rd^) fascicle. In wt conditions, these FasII-positive axons do not cross the midline. Examination of the commissural and longitudinal pathways labelled by HRP and anti-FasII in the VNC of *Syx1A* mutants revealed specific defects in diverse axonal pathways. From stage 14 onwards, by analyzing FasII-positive axons, we detected guidance defects in the VNC of 50% of the embryos (n = 38). These defects included aberrant midline crossing, as well as abnormal arrangement of ipsilateral fascicles (Fig 1D, 1E and 1F). Of these embryos, 18% displayed strong defects (Fig 1D and 1F) and 32% weak defects (Fig 1E and 1F, see Materials and Methods for quantification methods and definition of weak and strong phenotypes). FasII-positive fibers never crossed the midline in wt conditions. In contrast, Syx1A embryos displayed clear abnormal crossing of FasII-positive axons, thereby suggesting that longitudinal fibers aberrantly cross the midline in Syx1A mutants (Fig 1G). When HRP staining (a pan-axonal marker) was used, control embryos presented regularly spaced commissures (Fig 1C and 1H). However, embryos with the strong Syx1A phenotype showed collapsed commissures with no clear separation between anterior and posterior ones (Fig 1D and 1H). We analyzed and quantified commissural phenotypes in the VNC of *Syx1A* embryos displaying phenotypes (weak and strong phenotypes, 50% of embryos). Collapsed commissures (98% Fig 1H, arrows, and 1I) and “fuzzy” commissures were detected in many segments (36%, Fig 1H, asterisk, and 1I), as well as thinning of longitudinal fascicles between segments (57%, Fig 1H, arrowhead, and 1I).

On average, *Syx1A* mutant embryos displayed more than one defect in VNC axonal guidance per embryo (Fig 1G). Among these defects, the strongest were axonal midline crosses (Fig 1D, short arrow, 13%), commissural thinning (Fig 1H and 1I, 55%), and thinner longitudinal fascicles (Fig 1H and 1I, 32%), which were not detected in controls. The most penetrant phenotypes were fasciculation defects (Fig 1E, long arrow, 58%), but *Syx1A* mutant embryos also showed fascicle collapse (Fig 1D and 1E, arrowhead, 42%). Variability among these axonal phenotypes between individuals possibly reflects the differential contribution of the maternally deposited gene product. These results show that loss of *Syx1A* function induces axonal guidance defects in both commissural and longitudinal axons at embryonic stages of fly CNS development.

### *D*. *melanogaster nSyb*, *Snap25*, and *Vamp7* mutants display axonal guidance defects

**Fig 2:**
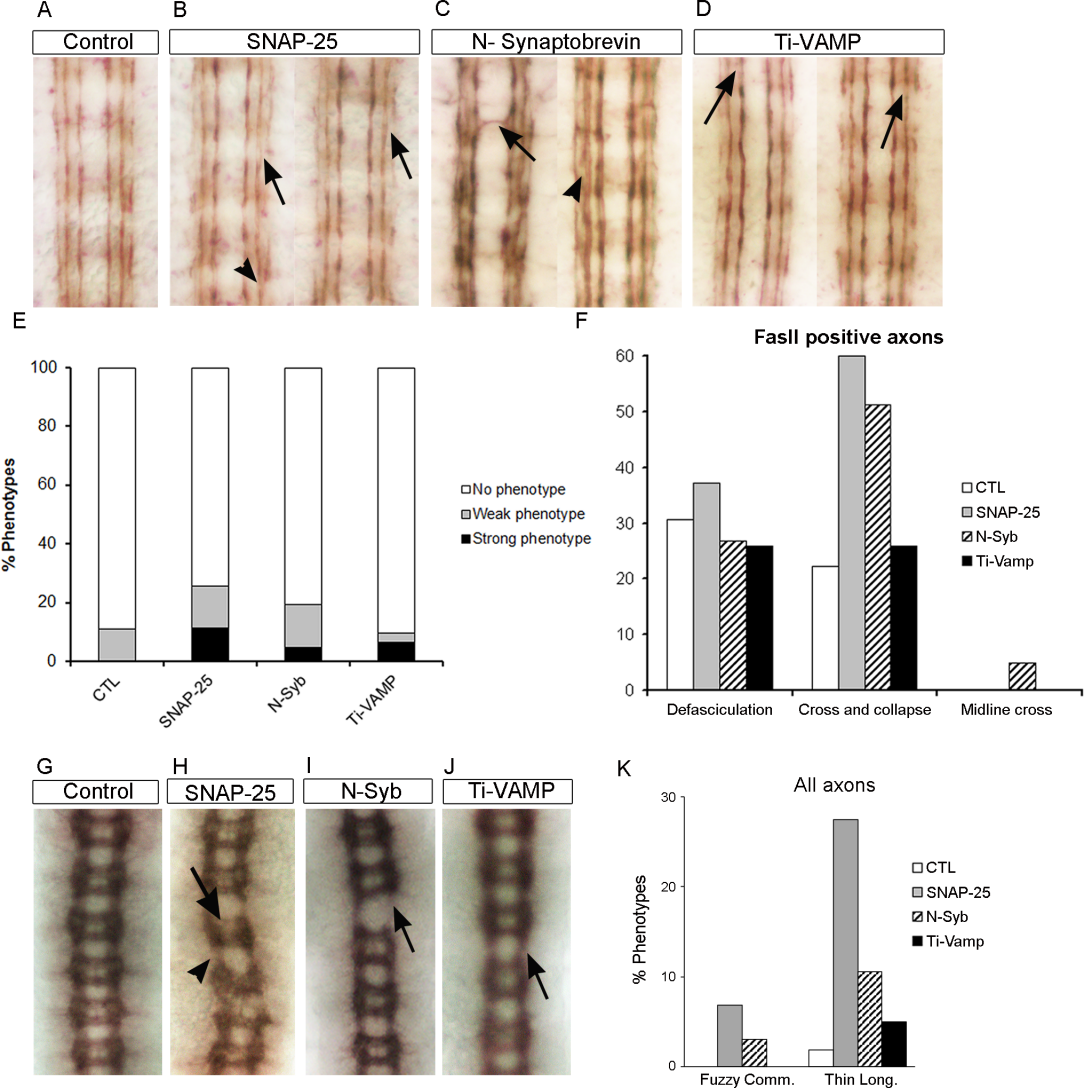
Diverse axonal pathway defects encountered in the VNC of *n-syb*, *snap-25*, and *Ti-VAMP* mutant embryos. (A-D) Stage 16 embryos were stained with anti-FasII to mark all FasII-positive axons and to better observe longitudinal axonal pathway defects. A, wt embryo showing the three FasII-positive longitudinal connectives. B, *SNAP-25* mutant embryos, representative of defasciculation (arrow) and fascicle collapse (arrowhead) phenotypes. C, *nSyb*^*d02894*^ mutant embryos, representative of midline crosses (arrow) and defasciculation phenotypes (arrowhead). D, *Vamp7* mutant embryos showing mild defasciculation phenotypes (arrow). (E) Quantification of the number of axon guidance defects encountered in *nSyb*, *SNAP-25*, and *Vamp7*/*Ti-VAMP* mutants in comparison with the background defects in the wt.(F) Quantification of the diverse axonal guidance defects encountered in *n-syb*, *Snap-25*, and *Vamp7/Ti-VAMP* mutants in comparison with the background defects in the wt. (G-J) Stage 16 embryos were stained with BP102 antibody to mark all axons in the VNC. G, wt embryo showing a detail of 6 segments with its anterior and posterior commissures. H, *Snap25* mutant embryos, representative of fuzzy commissures (arrow) and thinning of longitudinals (arrowhead) phenotypes. I, *nSyb*^*d02894*^ mutant embryos, representative of thinning of longitudinals (arrow). J, *Vamp7* mutant embryos showing mild thinning of longitudinals phenotype (arrow). (K) Quantification of the two axonal guidance defects encountered in SNAP*-25*, *n-syb* and *Vamp7/Ti-VAMP* mutants in comparison with the background defects in the wt.

We next used the same approach to study whether genetic loss of additional components of the SNARE core complex (SNAP25, VAMP2 and Ti-VAMP/VAMP7) also alters the commissural and longitudinal pathways in *D*. *melanogaster*. To do so, we examined mutants for *nSyb* (human VAMP2 ortholog) [40] and *Snap25* [41], and analyzed the *D*. *melanogaster* ortholog of Ti-VAMP/VAMP7 (*Vamp7* source:Flybase). Overall, the *nSyb*, *Snap-25*, and *Vamp7* phenotypes were weaker than the *Syx1A* one (Fig 2A–2D). When analyzing FasII positive axons for fasciculation defects, the strongest axonal guidance phenotypes were found in *Snap25* mutants, in which 74% embryos displayed fascicle collapse, defasciculation, or both (Fig 2B and 2F, n = 35). 57% of *nSyb* mutants showed fascicle collapse, defasciculation, or both (Fig 2C and 2F, n = 41). In addition, a small percentage of *nSyb* embryos (4%, n = 41) showed axonal midline crosses of FasII positive axons (Fig 2C, arrow). *Vamp7* mutants exhibited the weakest phenotypes (Fig 2D and 2E n = 31). We could detect stronger phenotypes when all axons were visualized with BP102 antibody. Again, the strongest phenotypes were detected in *SNAP25* mutant embryos (Fig 2H and 2K) where 6.9% of embryonic segments showed fuzzy commissures and 27.5% showed thinning of longitudinal fibers (n = 80 segments). nSyb mutants displayed intermediate phenotypes, fuzzy commissures in only 3.1% of segments and thinning of longitudinal fibers in 10.6% (n = 80) (Fig 2I and 2K). In *Vamp7* embryos we could only detect thinning of commissures (in 5% of embryonic segments, n = 80) (Fig 2J and 2K). These results indicate that *Vamp7*, *nSyb*, and *Snap25* can also influence *D*. *melanogaster* axonal guidance at the midline, but to a lesser extent than *Syx1A*.

### Silencing of SNARE proteins alters midline guidance of commissural axons in the chick spinal cord

**Fig 3:**
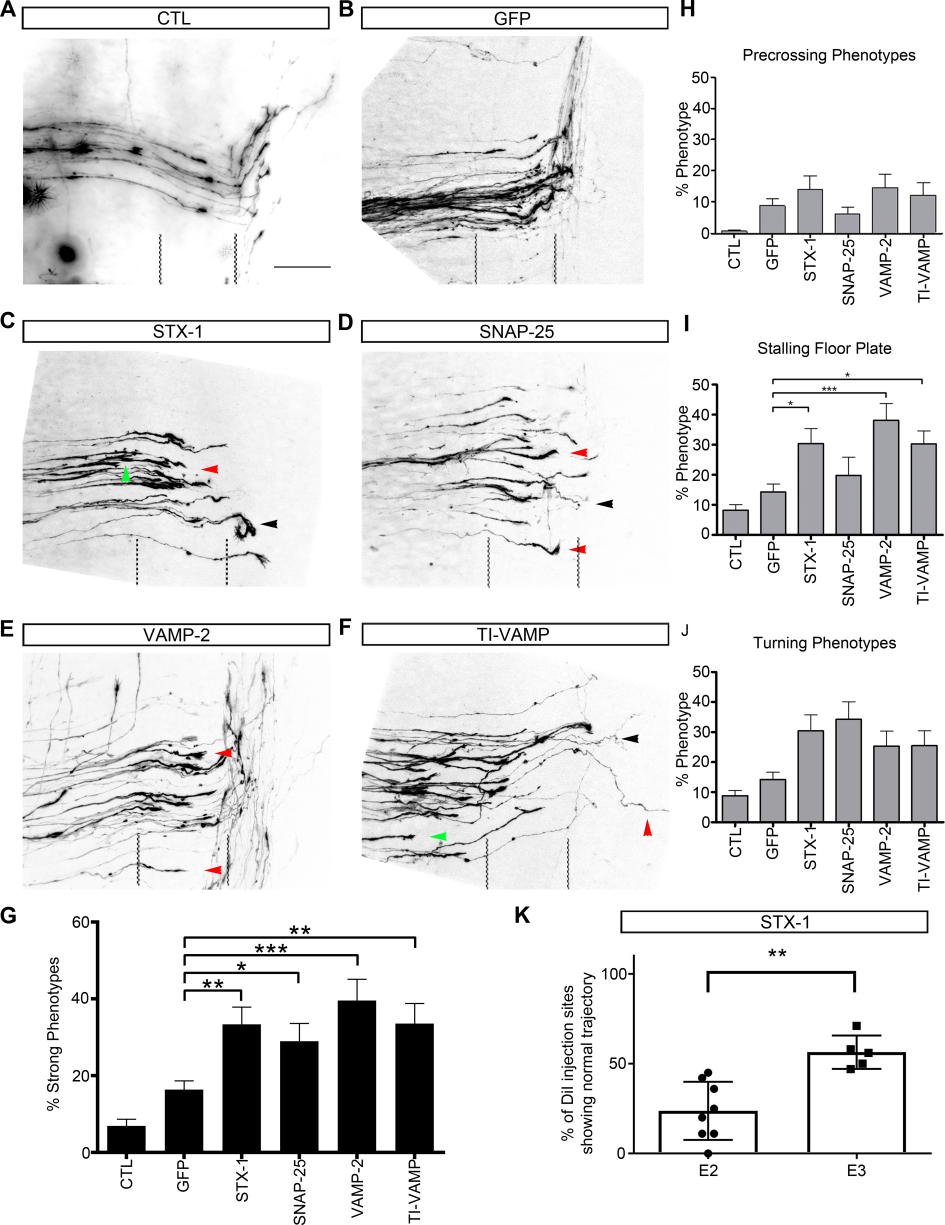
Silencing of SNARE proteins affected the guidance of commissural axons in chicken embryos. Commissural axons stained with the lipophilic dye DiI in open-book preparation of stage HH26 chicken embryos. In untreated control embryos (A) and in control embryos expressing EGFP (B) axons cross the midline and turn rostrally along the contralateral floor-plate border. In contrast, axonal tracing in embryos electroporated with dsRNA derived from STX-1A (C), SNAP-25 (D), VAMP-2 (E), or Ti-VAMP (F) revealed axonal stalling at the floor-plate entry site (green arrows), axonal stalling in the floor plate (red arrows), and aberrant or no turning into the longitudinal axis at the contralateral floor-plate border (black arrows). (G) After perturbation of SNARE signaling the percentage of DiI injection sites with normal axonal navigation per embryo was decreased: One-way ANOVA F5,226 = 11.99. p<0.0001. Newman-Keuls multiple comparison test: GFP-STX1 p<0.01; GFP-SNAP25 p<0.05; GFP-VAMP2 p<0.001; and GFP-TI-VAMP p<0.01. (H) Downregulation of individual SNARE proteins at E3 did not significantly enhance stalling at the floor-plate entry site. (I) In contrast, commissural axons failed to cross the floor plate after silencing SYT1A, VAMP2, or Ti-VAMP. In all these groups, axonal stalling in the floor plate was significantly increased in comparison to the EGFP-expressing control group. One way ANOVA F5, 199 = 8.232. p<0.0001. Newman-Keuls multiple comparison test: GFP-STX-1 p<0.05; GFP-SNAP25 p>0.05; GFP-VAMP2 p<0.001; and GFP-TI-VAMP p<0.05. Because a strong stalling phenotype (I) prevents independent analysis of the turning phenotype (J), ANOVA analyses were not done separately for the different phenotypes. Rather we compared the average percentage of DiI injection sites per embryo exhibiting aberrant axon navigation (G). When electroporation of dsRNA derived from STX1 was carried out already at E2 (K) the number of DiI injection sites per embryo with normal axonal trajectories was much lower compared to electroporation at E3. Scale bar in A: 50 μm.

Next, we used *in ovo* electroporation of double-stranded RNAs derived from STX1A, SNAP25, VAMP2, and Ti-VAMP to study the role of these genes in dI1 commissural axon guidance in the chicken spinal cord. In untreated controls and in EGFP-expressing control embryos, most commissural axons followed a stereotypic trajectory (Fig 3A and 3B). The vast majority of the dI1 axons crossed the floor plate and turned rostrally along the contralateral floor-plate border. See Methods for details on the quantification method. In contrast, we found that the down-regulation of all SNARE-complex proteins (STX1A, SNAP25, VAMP2, and Ti-VAMP) generated defects in commissural axon guidance, as axons either failed to enter or to cross the floor plate, or failed to turn into the longitudinal axis along the contralateral floor-plate border (Fig 3C–3G). After silencing STX1A aberrant axon pathfinding was found at 36% of the DiI injection sites per embryo (Fig 3C and 3G). We found overall similar percentages of injection sites with aberrant phenotypes when SNAP25, VAMP2 or Ti-VAMP were down-regulated (Fig 3G). When electroporated at embryonic day 3 (E3/HH17-18) most axons reached the floor plate and entered the midline area in all groups (Fig 3H). A detailed analysis of the aberrant phenotypes indicated a significant increase in floor-plate stalling in embryos where STX1A, VAMP2, or Ti-VAMP was silenced (Fig 3I). Similarly, turning of post-crossing axons was affected in all experimental groups (Fig 3J).

In a separate series of experiments, we compared electroporation of dsSTX1A at HH13/14 (E2) with electroporation at HH17/18 (E3) (Fig 3K). These experiments revealed an effect of timing of gene silencing on the severity of the phenotype. Electroporation at E3, when axons are starting to grow in the dorsal spinal cord, resulted in normal axon guidance at 56.4% of all DiI injection sites per embryo. In contrast, electroporation of neural precursors at E2 resulted in normal axon navigation only at 23.8% of all DiI injection sites. Importantly, failure of some axons to enter the floor plate was observed in 3/8 embryos electroporated early but was never observed in the embryos electroporated late. These data support the notion that the silencing of SNARE proteins in the chicken spinal cord leads to various commissural axon guidance defects.

### STX1A/B mutant mice present axonal guidance defects in the spinal cord

**Fig 4:**
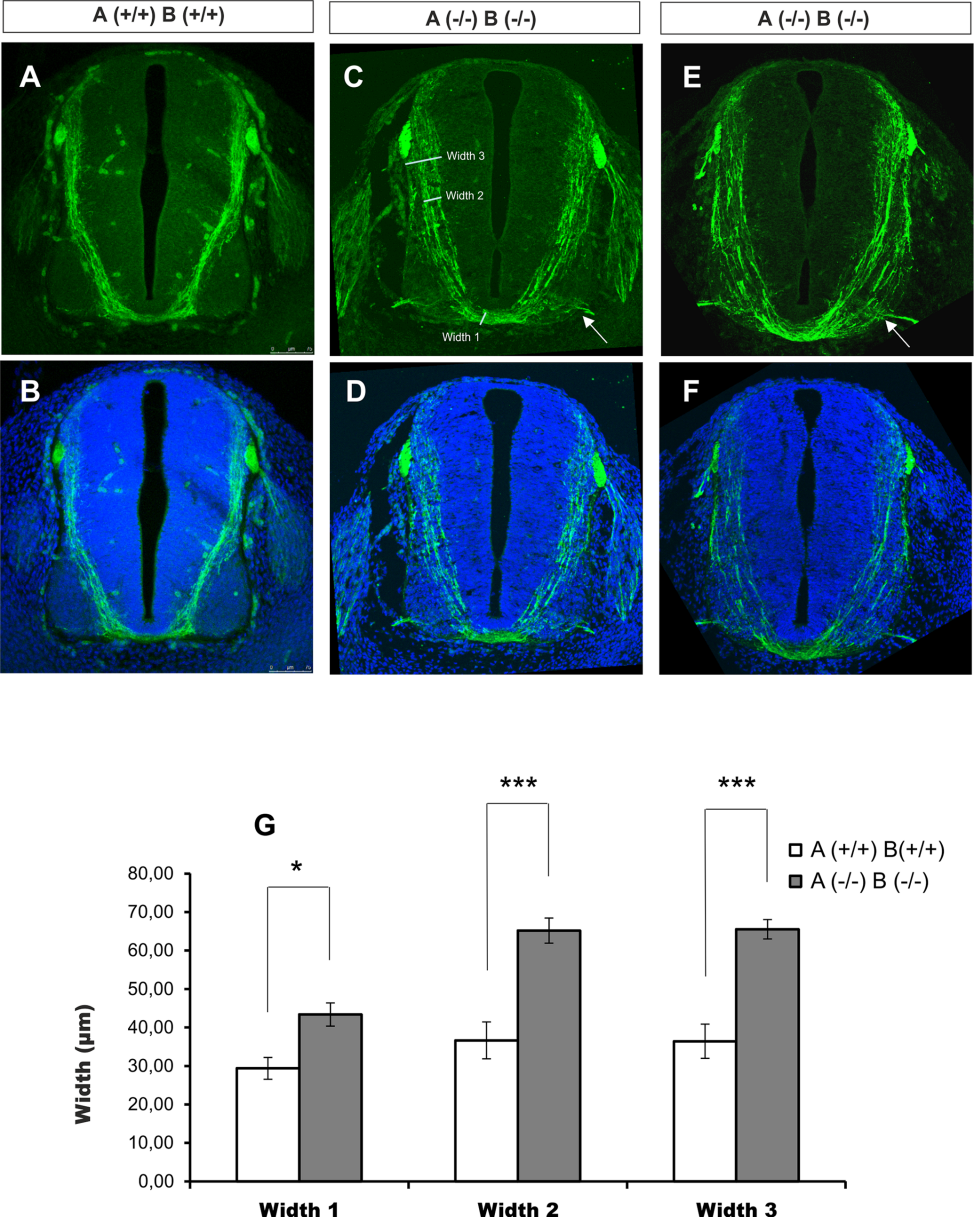
Syntaxin1 A/B mutant mouse embryos have aberrant axonal guidance phenotypes. (A-F) E12 mouse spinal cords; commissural axons immunostained with α-TAG-1 (A, C and E) and superposition with DAPI staining (B, D and F). (A) and (B) correspond to wt genotypes. The double KO genotype for STX1 (C-F) results in aberrant axonal guidance towards the floor plate. Axons are defasciculated and invade the motor column but still enter the floor plate. Arrows point at commissural axon bundles leaving the spinal cord ectopically through the motor exit point. (Scale bar: 75 μm). (G) Histogram illustrating the axon bundle width at three levels of the transversal section of the spinal cord: width 1, width 2 and width 3 were measured in wt and KO spinal cords. Significant differences are labelled by asterisks (*p≤0.05), (***p≤0.001). In (C) an example of the measurements taken for the quantification of the commissural bundle width is shown. Data are presented as the mean ± SEM. Statistical significance was determined using two-tailed Student’s t-test. Differences were considered significant at p<0.05.

To study the involvement of STX1 in commissural axon guidance in mammals, we generated double KO mice for the two STX1 isoforms, STX1A and STX1B. Double KO embryos died just after birth and displayed strong motor abnormalities, thereby suggesting severe alterations in nervous system organization. To address axonal phenotypes in the midline, we examined the commissural pathway in the spinal cord in E12 embryos [42,43]. In wt embryos, commissural axons stained with TAG-1 antibodies were organized as a narrow axonal bundle extending from the dorsal spinal cord towards the floor plate without invading the motorneuron area (Fig 4A and 4B). In contrast, in STX1A/B (-/-) mice, TAG-1-positive commissural axons were still directed towards the ventrally located floor plate, although their organization differed from that of controls. Instead of forming a narrow bundle, axons in STX1A/B (-/-) mice were clearly defasciculated, invading the entire mantle zone. Individual fibers or bundles invading lateral motorneuron areas were frequently observed (Fig 4C–4F). However, axons appeared to reach the floor plate. To support these observations, we quantified the width of the commissural pathway at three dorso-ventral levels (Fig 4C–4G). At each one, we found a significant difference in the width of commissural axon bundles between wt and double STX1 KO mice.

To confirm these findings, we stained spinal cord sections with Robo3 antibodies, a marker of pre-crossing and post-crossing commissural fibers. In wt embryos, Robo3-stained fibers formed a tight fascicle directed towards the floor plate. Moreover, post-commissural fibers in the ventral spinal cord were heavily stained (S2A and S2B Fig). In contrast, commissural fibers in STX1A/B (-/-) embryos exhibited wider ipsilateral fascicles, often invading the lateral (motorneuron) domains in the ventral spinal cord. Many ipsilateral commissural axons in mutant embryos were tipped with growth cones indicating that they failed to reach the floor plate. Consistent with this observation, the bundles of post-crossing commissural axons (located in the ventral spinal cord) were markedly decreased in STX1A/B null-mutant embryos (S2C and S2D Fig). Taken together, our observations indicate that the lack of STX1 results in aberrant commissural axon growth on both ipsilateral and contralateral sides.

**Fig 5:**
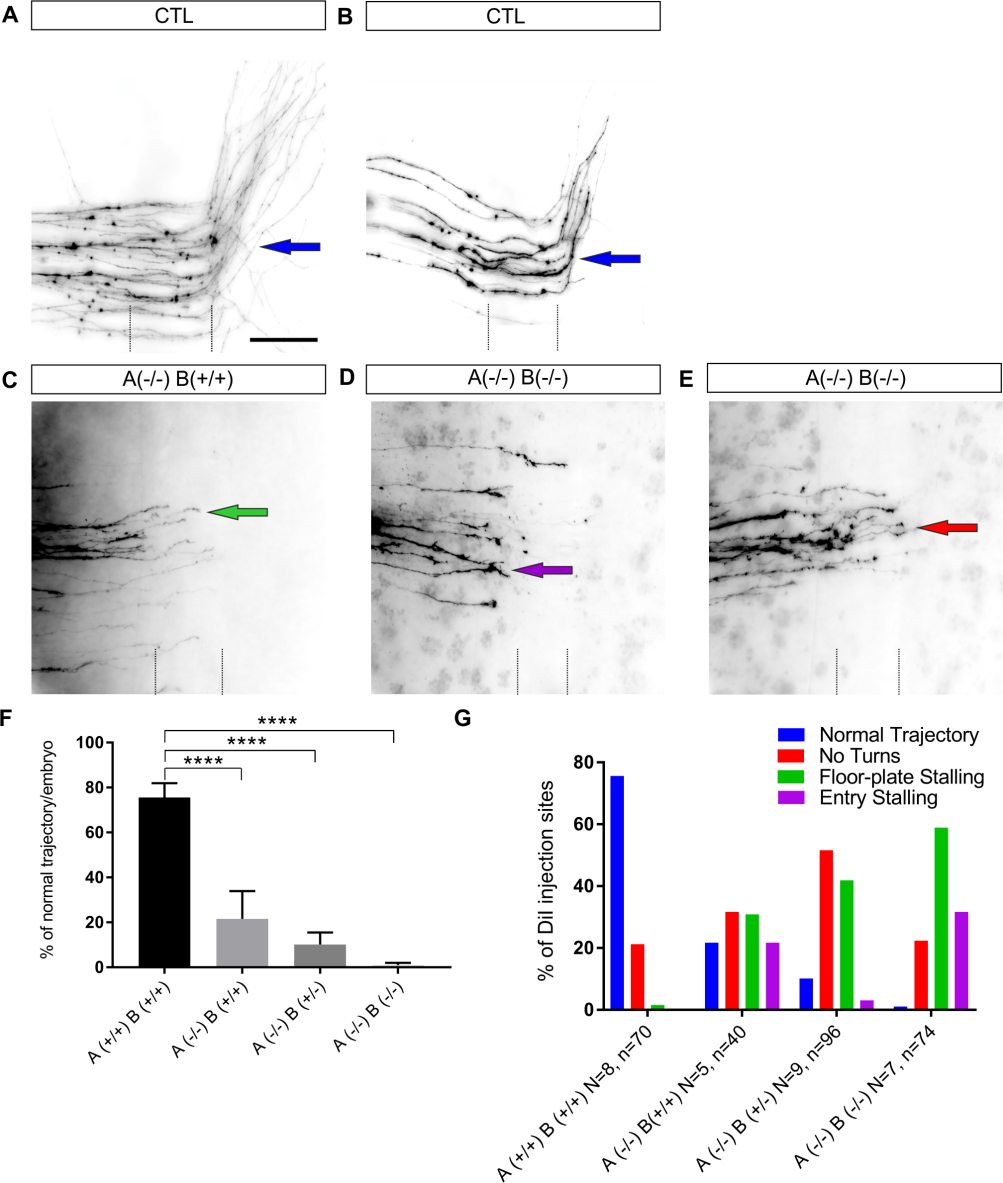
Commissural axon navigation in open-book preparations of mouse spinal cords. (A,B) Tracing of dI1 commissural axons in open-book preparations of spinal cords dissected from E12 wild-type embryos revealed axonal extension towards the floor plate, midline crossing and rostral turning along the contralateral floor-plate border. In general, we found more labeled axons per DiI injection site in wild-type compared to mutant embryos. We added a second image from a wild-type embryo with fewer labeled axons (B) for direct comparison with the mutant embryos (C-E). (C) In mutant embryos lacking both alleles of STX1A, axons extended towards the midline but failed to enter or stalled within the floor plate (green arrow). Axons that manage to reach the contralateral floor-plate border mostly failed to turn. (D,E) These aberrant phenotypes were even seen more often in double KO embryos. Furthermore, we also found more DiI injection sites where fibers failed to enter the floor-plate area in double KO embryos (G). (F) The quantitative analysis of normal trajectories in the different groups indicated significant differences between all mutant groups compared to wild-type mice: normal trajectories in wild-type at 75.6±5.9% (n = 8, 70 injection sites) of the DiI injection sites compared to 21.7±11.1% in STX1A^-/-^/STX1B^+/+^ (n = 5; 40 injection sites) and 10.2±5.4% in STX1A^-/-^/STX1B^+/-^ (n = 9; 96 injection sites) mice. Only 1.0±0.9% of the injection sites were normal in double knock-out mice (n = 7; 74 injection sites). **p<0.01, ***p<0.001, ****p<0.0001 compared to wild-type. (G) The detailed analysis of the individual guidance steps demonstrated mainly floor-plate stalling (green arrows) and failure to turn rostrally into the longitudinal axis (red arrow) as navigation defects in mutant mice. At many injection sites axons were unable to enter the floor plate (purple arrow). Bar: 70 μm.

To confirm the above observations, we also examined commissural axon navigation in open-book preparations of E12 spinal cords (Fig 5). Comparable to our observation in the chicken spinal cord, we found aberrant navigation of dI1 axons at the floor plate. In the double STX1 KO embryos, almost no axons were found to cross the midline and to turn properly along the contralateral floor-plate border (Fig 5D and 5E). Axons mainly failed to reach the contralateral border of the floor plate. In embryos lacking STX1A but expressing STX1B from one or two wt alleles, axon guidance was still compromised, exhibiting intermediate phenotypes (Fig 5C, 5F and 5G). A quantitative analysis showed a pronounced decrease in normal axonal trajectories in mutant compared to wt embryos (Fig 5F). In wild-type mice we found normal axon trajectories at 75.6±6.3% of the DiI injection sites per embryo. In contrast, in mice lacking STX1A but expressing one or two wild-type allele(s) of STX1B normal trajectories were only seen at 10.2±5.4% or 21.7±12.3% of the DiI injection sites, respectively. In double KO mutants axon navigation was affected even more strongly, as normal trajectories were only seen at 1% of the injection sites (Fig 5F and 5G). A detailed analysis of the different guidance defects revealed a problem in floor-plate stalling in all mutants in addition to a failure to turn rostrally along the contralateral floor-plate border. Failure to enter the floor plate was only found in mutants but never in wild-type mice (Fig 5G). Embryos having at least one wt allele of either STX1A or STX1B exhibited weaker axonal defects than the double KO mice (Fig 5G).

### Syntaxin 1 is required for axonal sensitivity to Slit-2 and Netrin-1

**Fig 6:**
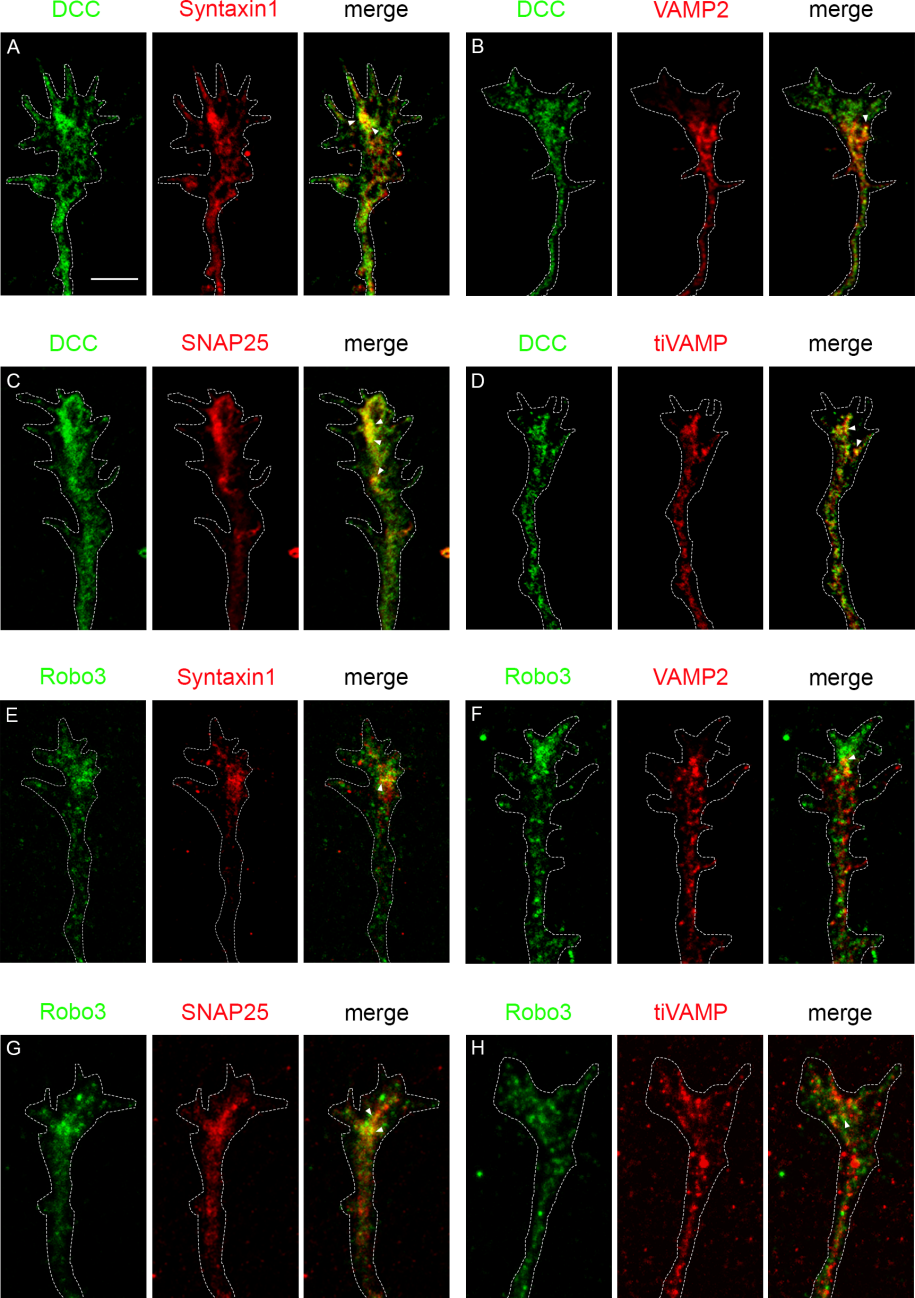
Colocalization of SNARE proteins and DCC/Robo3 in mouse commissural axons. Dissociated mouse commissural neurons were stained for different combinations of DCC and Robo3, and SNARE proteins (Syntaxin1, VAMP2, SNAP25 and Ti-VAMP). Analyses of confocal images from growth cones revealed partial colocalization between: (A) DCC and Syntaxin1; (B) DCC and VAMP2; (C) DCC and SNAP25; and (D) DCC and Ti-VAMP. Colocalization was also found between (E) Robo3 and Syntaxin1; (F) Robo3 and VAMP2; (G) Robo3 and SNAP25; and (H) Robo3 and Ti-VAMP. Arrowheads point to areas of colocalization. Scale bar: 5 μm.

To confirm the expression of SNARE proteins in spinal cord commissural axons we performed immunocytochemical analyses in neuronal cultures. Dissociated mouse commissural neurons were identified with two antibody markers: DCC and Robo3 antibodies. Cultures were co-immunostained for different SNARE proteins including STX1A, VAMP2, SNAP25 and Ti-VAMP. Confocal images revealed that commissural growth cones, identified by the expression of DCC and Robo3, co-expressed all the studied SNARE proteins (Fig 6). We also observed variable degrees of colocalization between DCC/Robo3 receptors and SNARE proteins. These results indicate that embryonic commissural axons express the SNARE proteins analyzed in the present study.

**Fig 7:**
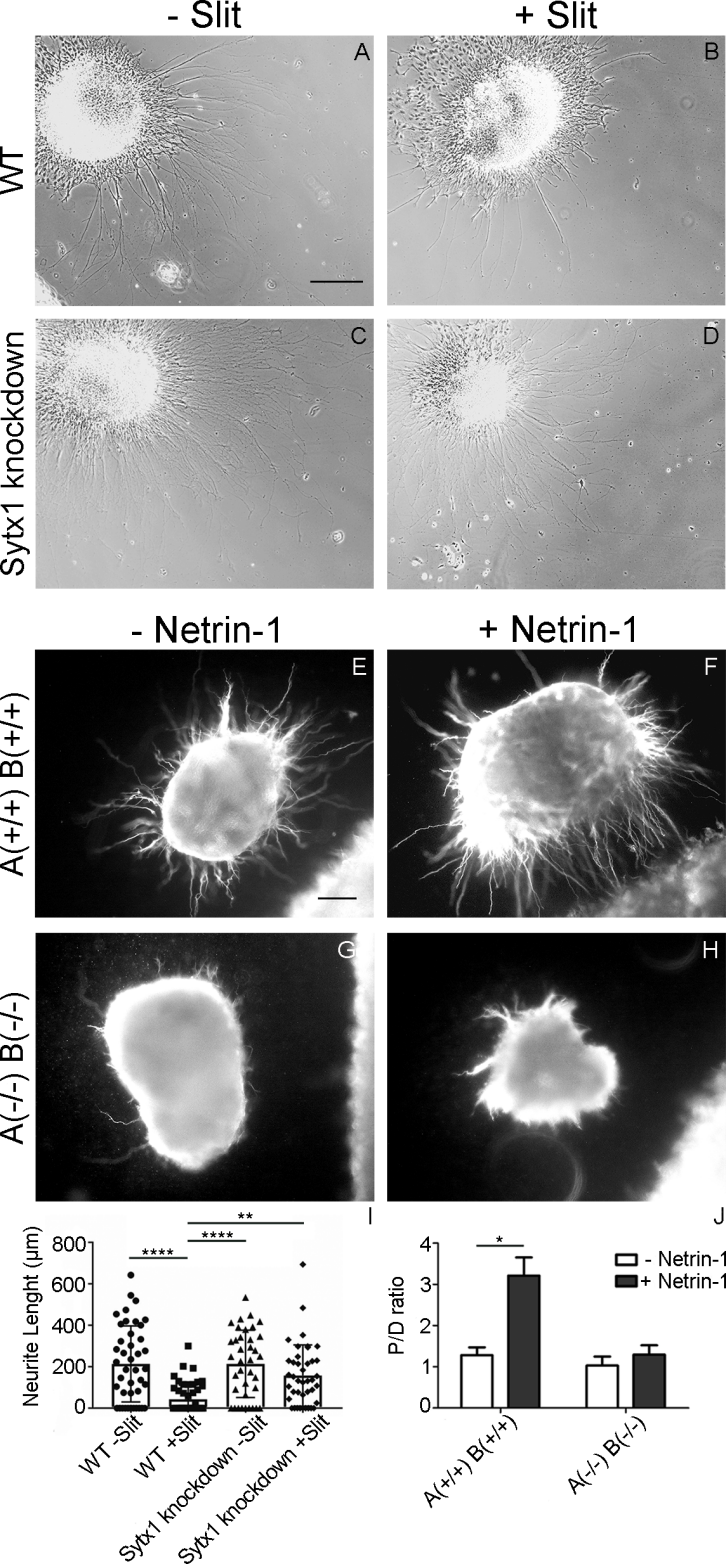
Syntaxin1A is required for axonal sensitivity to Slit-2 and Netrin-1. (A-D) Explants of dI1 neurons dissected from chick embryos grown on laminin substrate extend neurites readily in the absence of Slit-2 (A). In the presence of Slit-2, neurite length is strongly reduced (B). In contrast, neurite growth from explants taken from embryos electroporated with dsRNA derived from STX1A did not differ in the absence (C) or presence (D) of Slit-2. Bar: 200 μm. (E-H) Dorsal spinal cord explants obtained from E11 wild-type and STX1A/B knock-out mouse embryos were confronted with HEK293T cells aggregates expressing or not Netrin-1. (E, F) Axons from wild-type explants showed a marked attraction when confronted to Netrin-1 expressing cell aggregates (F), in contrast to explants confronted with control cells which exhibited a radial axonal growth (E). Mutant spinal cord explants (STX1A(-/-)B(-/-)) exhibited a radial pattern of axonal growth in all conditions (G, H). Scale bar: 100μm. (I) Plots showing average neurite lengths in explants of dl1 neurons incubated with Slit-2. Significant difference is exclusively seen in wild-type explants. (two-way ANOVA analysis; * p<0.05, ** p = 0.0011, ****p<0.0001). At least, 37 explants per condition were used for quantification. The average neurite length in control explants was 214 μm in the absence of Slit-2 (A,I; n = 42 explants) and 43 μm in the presence of Slit-2 (B,I; n = 51 explants). Explants taken from embryos after silencing STX1A extended neurites with an average length of 214 μm in the absence (C,I; n = 37) and 158 μm in the presence of Slit-2 (D,I; n = 41). J) Quantification of Proximal/Distal (P/D) ratios in spinal cord mouse explants confronted to Netrin-1 expressing cell aggregates (two-way ANOVA analysis; *p<0.05).

We next addressed whether STX1A is required for Slit-2 and Netrin-1 actions. Tissue explants of chick dorsal commissural neurons dissected from control embryos and grown on laminin are known to extend neurites readily in the absence of Slit-2 (Fig 7A). In the presence of Slit-2, neurite length was strongly reduced (Fig 7B). In contrast, neurite growth from explants taken from embryos electroporated with dsRNA derived from STX1A did not differ in the absence or presence of Slit-2 (Fig 7C, 7D and 7I), indicating that the absence of STX1A results in a markedly reduced Slit-2 response of chick commissural axons.

We have previously shown that the blockade of STX1 with Botulinum Toxin C1 reduces Netrin-1 induced chemoattraction in mouse spinal cord explants [16]. To confirm this finding, we co-cultured in collagen gels dorsal spinal cord explants from STX1 KO embryos with Netrin-1 expressing cell aggregates. In comparison with explants cultured with control cells (exhibiting radial growth, Fig 7E), wild-type explants confronted to aggregates of Netrin-1 expressing cells showed strong chemoattraction (Fig 7F and 7J). In contrast, Netrin-1 induced chemoattraction was absent in spinal cord explants derived from double STX1A/B KO embryos (Fig 7G, 7H and 7J).

Our previous studies have shown that exposition to Netrin-1 increases DCC surface expression in growth cones and that such increase is not diminished after blockade of STX1 [15,16]. Here we performed experiments to determine whether STX1 regulates Robo3 surface expression in the presence of Netrin-1. Mouse embryonic commissural neurons were cultured and stained for the differential immunolabeling of surface and intracellular Robo3 protein pools (S3 Fig). The data show that in control commissural growth cones, Robo3 trafficking and distribution is not substantially altered by Netrin-1 incubation. In contrast, STX1A/B knock-out growth cones incubated with Netrin-1 exhibited a decrease in surface Robo3 signals (S3D and S3E Fig). These findings suggest that the inactivation of STX1 alters Robo3 trafficking and/or surface expression, probably by increasing Robo3 internalization.

### *Drosophila* Syx1A interacts genetically with the Slit/Robo and Netrin-1/DCC pathways

**Fig 8:**
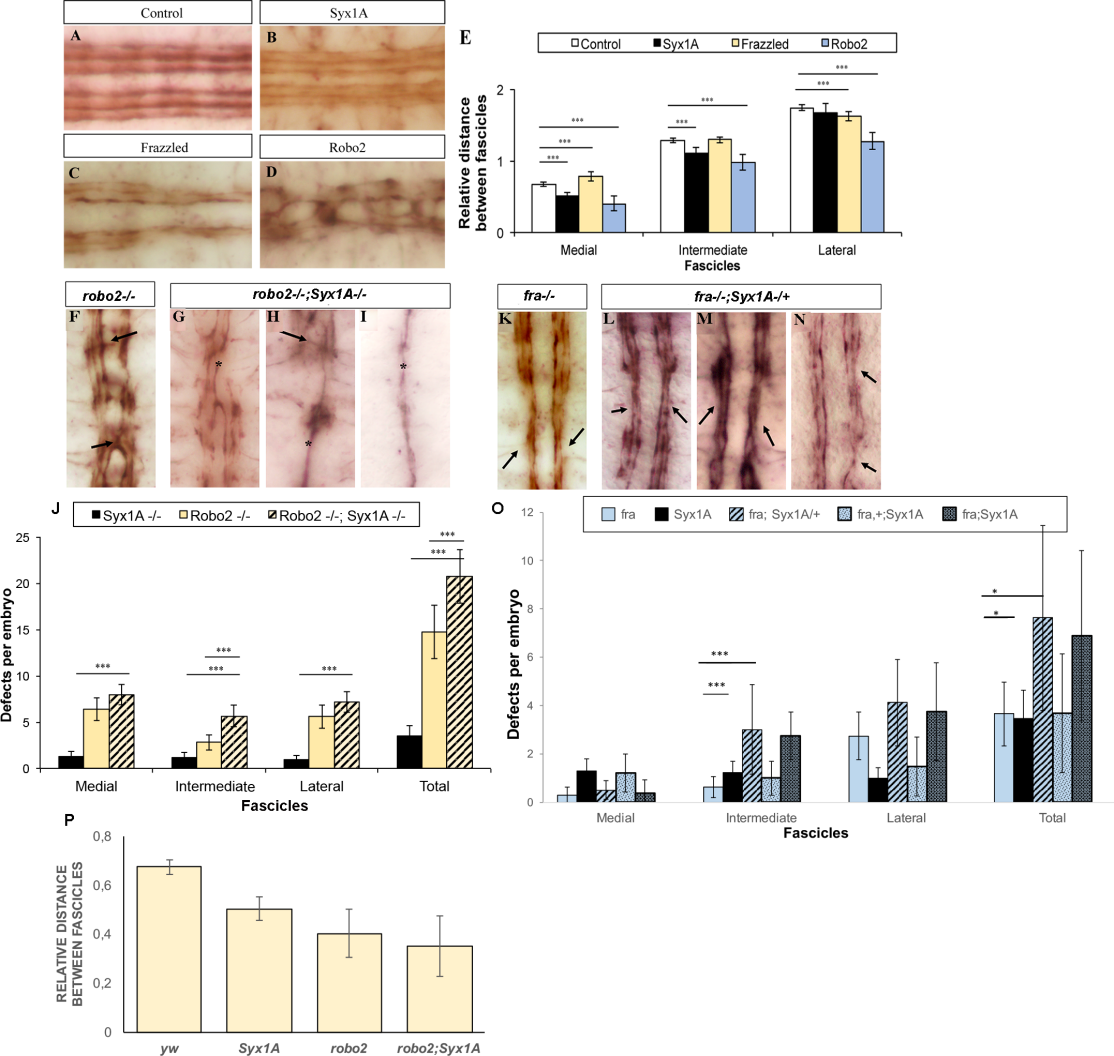
*Drosophila Syx1A* genetically interacts with to *robo2* and *fra*. (A-D) Stage 16 embryos stained with anti-FasII to mark all FasII-positive axons. A, wt embryo showing the three FasII-positive longitudinal connectives. B, *Syx1A* mutant embryo showing the three longitudinal connectives closer to the midline. C, *fra*^*3*^ mutant embryo showing an increased distance between fascicles. D, *robo2* mutant embryo showing midline crosses and fascicles closer to the midline. (E) Quantification of the relative distance between fascicles in control (n = 20), *Syx1A* (n = 20), *fra* (n = 37) and *robo2* (n = 20) VNCs. Significant differences are labelled by asterisks (***p≤0.001). (F-I) Stage 16 embryos stained with anti-FasII to mark all FasII-positive axons and to better observe axonal pathway defects. F, *robo2* mutant embryo showing midline crosses (arrows) and fascicle collapses. G-I, *robo2;Syx1A* double mutant embryos representative of the three phenotypes encountered; G: Weak, H: Intermediate and I: Strong. Asterisks show fascicle collapses in the VNC in all cases. Anterior is up. (J) Quantification of the total number of axon guidance defects encountered in *Syx1A* (n = 38), *robo2* (n = 39) and *robo2;Syx1A* (n = 31) mutant embryos. Significant differences are labelled by asterisks (***p≤0.001). (K-N) Stage 16 embryos stained with anti-FasII to mark all FasII-positive axons and to better observe axonal pathway defects. K, *fra*^*3*^ mutant embryo showing fascicle defasciculation and collapse phenotypes (arrows). L-N, *fra;Syx1A/+* mutant embryos representative of the phenotypes encountered. Arrows point at defasciculation and collapsed phenotypes. (O) Quantification of the total number of axon guidance defects encountered in *fra* (n = 37), *Syx1A* (n = 31), *fra/+;Syx1A* (n = 15), *fra;Syx1A/+* (n = 8) and *fra;Syx1A* (n = 8) mutant embryos. Significant differences are labelled by asterisks (*p≤0.05,***p≤0.001). (P) Quantification of the relative distance between medial fascicles in control (n = 20), *Syx1A* (n = 20), *robo2* (n = 20) and *robo2;Syx1A* (n = 17) VNCs. Only accounted the cases like panel G, where fascicles had not collapsed.

During our analysis of midline guidance defects in *Syx1A* mutant embryos, we detected that the arrangement of the longitudinal fascicles was shifted towards the CNS midline compared to controls (Fig 8A and 8B; n = 20). In order to clarify whether the FasII–positive axonal fascicles were closer to the midline in *Syx1A* mutants, we quantified these distances at embryonic stage 16 and compared them to wt, *frazzled* (*fra*) and *robo2* mutants (Fig 8C–8E). Overall, *Syx1A* and *robo2* mutations induce a shift of FasII-positive fibres (both FasII-m and FasII-i) towards the midline. Regarding both FasII-m and FasII-I, the opposite occurs in *fra* mutant embryos, thereby suggesting that Syx1A interferes with a midline repression pathway (Fig 8A–8E) [44].

In order to address whether Syx1A is involved in the Slit/Robo pathway, we generated double mutants for *Syx1A* and *robo2* and examined the positioning of the longitudinal tracts in *robo2;Syx1A* embryos. As shown in Fig 8F–8J, the longitudinal fascicle distance to the midline decreased in the double mutant embryos (Fig 8I and 8P), where the strongest phenotypes showed a complete collapse of the tracts in the midline (Fig 8I, asterisk), as reported for *slit* (*sli*) mutants [45]. This result indicates that S*yx1A* and *robo2* interact genetically. In molecular terms, our above results suggest that Syx1A is involved in axonal repulsion in the midline.

In vertebrates, it has been reported that Netrin1/DCC axonal guidance is coupled to exocytosis through STX1 [15]. Therefore, we examined whether differences in Syx1A levels could affect the *Drosophila fra* axonal guidance phenotypes. We found that the *fra* mutant phenotype was significantly aggravated by removing one copy of *Syx1A* (Fig 8K–8O), thereby suggesting that Syx1A levels severely interfere with the Netrin/Fra guidance pathway.

## Discussion

Here we used loss-of-function models in three species (fly, chick and mouse embryos) to examine the role of SNARE proteins in midline crossing of commissural axons. We report aberrant axonal phenotypes in the *D*. *melanogaster* midline and in the chicken spinal cord. Furthermore, we generated double mutant STX1A/B mice that show abnormal commissural phenotypes in the murine spinal cord. These findings indicate an evolutionarily conserved role of SNARE complex proteins in midline axonal guidance.

The guidance of commissural axons at the midline of the spinal cord is a complex process regulated by many molecules. For instance, the interaction between Netrin-1 and DCC attracts pre-crossing axons toward the floor plate [42,43]. Axon growth towards the floor plate is also altered in mice lacking VEGF/Flk1 signaling [46]. Midline crossing was shown to depend on attractive (Axonin-1/TAG1 with NrCAM; [47]) and repulsive interactions (Slit/Robo; [48]; Sema3B/NrCAM/PlexinA1/Neuropilin; [49] [50] [51,52]). The rostral turn of post-crossing commissural axons was shown to depend on morphogen gradients formed by Wnts [53,54] and Shh [55,56], although the latter is also involved in attraction of commissural axons toward the ventral midline in parallel to Netrin-1 [57,58]. In addition, an interaction between axonal Semaphorin6B and its ligand PlexinA2 [59] and interactions between the SynCAMs [60,61] are also involved in the turning of post-crossing commissural axons into the longitudinal axis.

Our results in *D*. *melanogaster* indicate that the SNARE complex is involved in midline axonal guidance. We show that *Ti-Vamp*/*Vamp7*, *nSyb*, and *Snap25* influence axonal guidance at the midline, but to a lesser extent than *Syx1A*. In general, these phenotypes may be incomplete *Drosophila* KOs, because in this fly there is a maternal contribution for all these components. Additionally, it is known that there is a high level of redundancy in *D*. *melanogaster*. In this regard, it has been reported that *Syb* can replace *nSyb* and *Snap24* can replace *Snap25* [40,41]. Regarding the *Syx1A* phenotypes, we can conclude that *Syx1A* loss of function induces commissural and ipsilateral axonal phenotypes. Interestingly, some of the phenotypes observed in *Syx1A* mutants resemble the loss of function phenotypes of the Robo/Slit pathway [62,63]. We detected midline crosses of FasII-positive axons and a strong genetic interaction with *robo2*. These findings suggest that Syx1A is involved in repulsive midline guidance in *Drosophila*. In addition, most of the ipsilateral phenotypes detected are also common in *frazzled* (*fra*, the *D*. *melanogaster* homolog of DCC), and *fra* phenotypes are aggravated by decreased *Syx1A* levels. Furthermore, similar ipsilateral phenotypes to *Syx1A* are observed in *netrin* mutant ventral nerve cords [44], as well as in embryos mutant for Heparan Sulfate Proteoglycans (HSPGs) [64] and Hh [65], thereby suggesting that Syx1A may participate in various guidance pathways in *D*. *melanogaster* not just in axonal guidance but also in fasciculation/defasciculation events. Recent studies suggest that whereas axonal attraction requires exocytosis, chemorepulsion relies on endocytosis [14,18,66,67]. In addition, SNARE proteins have been reported to be involved in both exo- and endocytosis [68]. Our genetic interaction experiments support the notion that Syx1A participates in both Netrin/Fra attraction and Slit/Robo repulsion.

The data obtained in chicken reinforce the idea that the silencing of SNARE proteins induces an overall increase in various commissural guidance defects, with the silencing of STX1A leading to defects in all the guidance steps analyzed. Silencing STX1A at E2, on day before neurons start to extend axons rather than E3, just before they start to extend axons, also resulted in axons failing to enter the midline area. This phenotype resembled previously reported findings on the role of STX1A in DCC-mediated guidance of pre-crossing commissural axons towards the midline [16].

Based on these results, we conclude that SNARE proteins make a crucial contribution to the navigation of chick commissural axons at the floor plate. SNARE proteins are involved in both attractive [16] and repulsive (this study) decision-making steps in axon guidance, as STX1A, VAMP2 and Ti-VAMP silencing leads to a substantial increase in pre-crossing and post-crossing phenotypes. These phenotypes are in agreement with those reported in previous studies [15,16], confirming the involvement of STX1 in the regulation of chemoattractive guidance pathways for commissural axons.

The phenotypes seen in the chicken spinal cord resemble those observed after silencing Calsyntenin-1 and RabGDI in dI1 neurons [69]. In the absence of Calsyntenin-1 and RabGDI, Robo1 is not transported to the growth cone surface, resulting in axonal stalling in the floor plate. Furthermore, silencing Calsyntenin-1, but not RabGDI, prevents the expression of the Wnt receptor Frizzled-3 on the growth cone surface, leading to failure of post-crossing axons to turn rostrally in response to the Wnt gradient [54]. These findings are consistent with the analyses of commissural axon navigation at the midline in STX1 KO mice. In double mutants, the absence of STX1A and STX1B proteins prevented midline crossing.

In Netrin-1 [70,71] and DCC KO [44,70] mice, pre-commissural fibers display strong phenotypes, including defasciculation, aberrant trajectories, and a complete failure to cross the floor plate. Weaker phenotypes have been observed in mouse mutants for other genes involved in commissural axonal guidance, including Sonic Hedgehog and the VEGF/FlK1 pathways [46,57]. The phenotype described here in STX1A/B mutant mice is reminiscent of, that in Netrin-1 and DCC KO mice, with fewer axons reaching the floor plate, lateral invasion of motorneuron territories, and fewer post-crossing commissural axons, although the phenotype is much weaker and many fibers still reach the ipsilateral floor-plate border. A likely explanation could be that although STX1A/B might be required for correct sensing of Netrin1/DCC guidance [15,16], compensatory mechanisms may derive from the possible lack of STX1A/B requirement in other commissural attractive pathways (e.g., SHH or VEGF/Flk1). Our findings indicate that STX1A/B not only affects pre-crossing axons but strongly affects midline crossing and post-commissural axonal guidance. This conclusion is supported by our findings in *Drosophila*, which revealed a genetic interaction between *Syx1A* and the Robo pathway, as well as by the present in vitro experiments showing that STX1 is required for both Netrin-1 attraction and Slit-2 repulsion (Fig 7).

Our previous studies showed that the lack of STX1 did not affect DCC surface expression induced by Netrin-1 [15]. In the present study we show that the lack of STX1 results in a decreased surface expression of the Robo3 receptor in response to Netrin-1. It is therefore possible that the lack of STX1 may alter the surface expression of other Robo family members (eg Robo1) and Frizzled-3, the receptors used by post-crossing axons. This idea is supported by our STX1 loss-of-function data, showing that the lack of this SNARE protein abolished the responsiveness of post-crossing commissural axons to Slit, both in vitro (explant experiments) and in vivo (chick and mouse analyses). We thus propose that, in contrast to DCC expression, the effect of STX1 loss-of-function on post-crossing axons may be explained in part by preventing the surface expression of axon guidance receptors required for midline crossing and post-crossing navigation.

Robo3 is an atypical Robo receptor that has been proposed recently to potentiate DCC-mediated attraction to Netrin-1, but without binding Slits [72,73]. Thus, the observation that Netrin-1 in STX1-deficient growth cones results in decreased Robo3 surface expression, supports the notion of a possible contribution of Robo3 membrane downregulation to explain the reduced Netrin-1 chemoattraction found in STX1 loss-of-function models.

Many guidance molecules and their receptors involved in axonal guidance are conserved between vertebrates and invertebrates (e.g. Robo, Hh, and Netrins) [57,65,74]. Our systematic analysis of the phenotypes at the CNS midline of fly, chick, and mouse embryos mutant for STX1 unveils an evolutionarily conserved role for STX1 in midline axonal guidance. Overall, the ipsilateral phenotypes reported are consistent with the participation of STX1 in Netrin-1-dependent axonal guidance, as proposed previously [15,16]. In addition, here we describe post-commissural phenotypes that are reminiscent of those found in Robo, NrCAM and VEGF loss-of-function models [46,47,75,76], thereby suggesting that STX1 underlies various signaling pathways. Furthermore, the phenotypes described herein for other SNARE proteins point to the participation of SNAP25, VAMP2 and Ti-VAMP in midline axonal guidance, although the precise implication and relevance of individual SNARE proteins to specific axonal guidance signaling complexes remains to be determined. We propose that the coupling of the guidance receptor cell machinery to proteins that regulate exocytosis is a general and conserved mechanism linking chemotropic signaling to membrane trafficking [29].

## Materials and methods

### *D*. *melanogaster* stocks and genetics

The following stocks are described in FlyBase (http://flybase.org): *Syx1A*^*Δ229*^, SNAP-25 (Df(3L)1-16), *nSyb*^*d02894*^, Ti-VAMP (P[CG1599^G7738^]), *fra*^*3*^ and *robo2*. All alleles used are genetic nulls. Wild-type control is *yw*. *D*. *melanogaster* stocks and crosses were kept under standard conditions at 25^º^C.

### Immunohistochemistry, image acquisition, and data analysis for all animal models

*D*. *melanogaster* embryos were staged as described by Campos-Ortega and Hartenstein [77] and stained following standard protocols. For immunostaining, embryos were fixed in 4% formaldehyde for 20 min. We used antibodies that recognize FasII (mAb1D4, DSHB), βGal (Promega), Affinity-Purified Anti-HRP TRITC (Jackson immunoResearch), and mAbBP102 (DSHB). We used biotinylated HRP (Amersham) or non-biotinylated HRP (GE Healthcare), Alexa488, Alexa-555 and Alexa-647, Cy2, Cy3 and Cy5 conjugated secondary antibodies (Jackson ImmunoResearch). For HRP histochemistry, the signal was amplified using the Vectastain-ABC kit (Vector Laboratories) when required. In addition, the signal for the DAB reaction was intensified with NiCl_2_, except for double stainings, where it was omitted from one of the reactions. DIC photographs were taken using a Nikon Eclipse 80i microscope. Fluorescent images were obtained with a confocal microscope (Leica TCS-SPE-AOBS and TCS-SP2-AOBS systems) and processed using Fiji [78] and Adobe Photoshop. Images are maximum projections of confocal Z-sections.

Fertilized chicken eggs were obtained from a local supplier, and embryos were staged following Hamburger and Hamilton [79]. Electroporations were performed either at HH13-14 (E2) or at HH17-18 (E3). Images were acquired using a confocal spinning disk microscope (Olympus BX61).

Mouse embryos aged 11 or 12 days (E11 and E12 respectively) were used for the experiments. To obtain the tissue samples, pregnant female mice were sacrificed by cervical dislocation. A small portion of the tail was cut for further genotyping. Embryos were then fixed with 4% paraformaldehyde, and spinal cord sections were immunostained with mouse anti—TAG1 (mouse, Hybridoma Bank), followed by α-mouse IgM biotinylated (goat, Chemicon) and by Streptavidine Fluorescent FITC (490 nm, GE Healthcare). Sections were routinely stained with bisbenzimide. Confocal images were acquired using a Leica TCS SP5 microscope with 20x and 40x oil-immersion objectives. An average of 4–6 embryos per genotype and 10–15 slices per embryo were analyzed. For the quantification of TAG1 staining we used 4 KO and 3 WT embryos (32 sections from KO embryos and 8 from WT embryos). Data are presented as the means ± SEM. Statistical significance was determined using two-tailed Student’s t-test. Differences were considered significant at p<0.05. Embryonic mouse spinal cord sections were also stained with goat α-Robo3 antibodies (1:100, RD Systems) followed by incubation with α-goat Alexa-488 secondary antibodies.

Experiments with chicken embryos were approved by the Cantonal Veterinary Office Zurich. All the experiments using animals were performed in accordance with the European Community Council directive and the National Institute of Health guidelines for the care and use of laboratory animals. Experiments were also approved by the local ethical committees.

### Quantification of fly axon guidance phenotypes

The percentage of axons displaying abnormal FasII phenotypes was quantified via anti-FasII stainings by an observer with no knowledge of the genotype. Defects were categorized as “midline crossing”, “defasciculation” or “fiber collapse”. Due to the variability of fasciculations and occasional fiber collapses in wt embryos, we allowed for up to 2 of these defects to be considered “normal” or “background” and therefore set our “zero” defects at this control level. From 2 to 5 defects, we considered that embryos had a weak phenotype, while with more than 6 defects they were considered to have a strong phenotype.

The percentage of axons displaying abnormal midline crossing phenotypes was quantified via anti-HRP or BP102 stainings by an observer with no knowledge of the genotype. Defects were categorized as “fuzzy commissures”, “collapsed commissures” or “thinning of longitudinals”.

Fascicle distances to the midline were quantified via anti-FasII stainings, where the distance between fascicles was measures. Distances were measured in arbitrary units, between media, intermediate and lateral fascicles and these values were normalized to the length of the axonal tracks in the VNC.

All data were analyzed statistically, SEMs were calculated and statistic significance assessed by Student’s t-test.

### *In vivo* analysis of commissural axon pathfinding in chick embryos

The analysis of commissural axon trajectories was performed as described previously [47,55]. In brief, fertilized eggs were windowed on the second or third day of incubation. Extra-embryonic membranes were removed to access the spinal cord *in ovo*. A plasmid encoding EGFP (20 ng/μl) and the dsRNA (250 ng/μl) derived from STX-1A, SNAP-25, VAMP2, or Ti-VAMP in PBS were injected into the central canal using glass capillaries. dsRNA was produced by *in vitro* transcription as described previously [80]. For the production of dsRNA derived from STX1A, SNAP25, VAMP2, or Ti-VAMP, we used ESTs obtained from Geneservices (now Source BioScience) [55]. For visualization and control of injection quality and quantity, 0.04% Trypan blue was added. For electroporation, we used platinum electrodes connected to a BTX square wave electroporator. Electrodes were positioned parallel to the longitudinal axis of the lumbosacral spinal cord of the chicken embryo, as detailed previously [55,81]. Five pulses of 26 V and 50 ms duration with a 1-s interpulse interval were applied. After electroporation, eggs were sealed with Scotch tape and incubated for another 2 or 3 days. The gene silencing specificity was verified by using two independent and non-overlapping sequences for the generation of the dsRNAs, when available. dsRNA sequences used in the present study were shown to downregulate the targeted proteins by 25%-66% [29]. Because in these conditions only about 60% of the cells are efficiently transfected, small decreases in the total amount of protein can still be indicative of efficient knock-down by dsRNA transfection.

For the analysis of commissural axon guidance, embryos were sacrificed between stages 25 and 26 [79]. The spinal cord was removed from the embryo, opened at the roof plate (“open-book” preparation), and fixed in 4% paraformaldehyde for 30–60 min.The trajectories of dI1 commissural axons at the lumbosacral level of the spinal cord were visualized by the application of the lipophilic dye Fast DiI (dissolved at 5 mg/ml in ethanol; Invitrogen) to the cell bodies. Care was taken to exclusively label the dorsal-most population of commissural neurons (dI1 neurons) to avoid confusion with more ventral populations that have distinct pathfinding behavior. Only DiI injections sites that were in the appropriate location in the dorsal-most part of the spinal cord and within the region expressing fluorescent protein were included in the analysis.

Quantification of the percentage of injections sites with axons displaying abnormal phenotypes was done by a person blind to the experimental condition. The injection sites were classified as ‘normal’ when the axons crossed the floor plate and turned rostrally along the contralateral floor-plate border. When at least 50% of the labeled fibers failed to reach the contralateral border of the floor plate, the DiI injection site was judged as ‘floor-plate stalling’, when at least 50% of the fibers reaching the floor-plate exit site failed to turn into the longitudinal axis, the DiI injection site was considered to exhibit ‘no turning’. Because stalling of all or most axons in the floor plate prevented the analysis of the turning phenotype, the quantification did not include a separate analysis of this phenotype between the different groups. In Fig 3G–3J, the average percentages of DiI injection sites per embryo with the respective aberrant phenotypes are shown.

### Generation of Syntaxin 1B KO mice

Mutant STX1B mice were generated using a gene-trapping technique [82]. Mice (strain C57BL/6 from Charles River Laboratories) were cloned from an ES cell line (clone OST68841; Texas Institute for Genomic Medicine, TIGM). The ES cell clone contained an insertion of the Omnibank Vector VICTR24 in the first exon of the STX1B gene identified from the TIGM gene trap database and was microinjected into C57BL/6 host blastocysts to generate germline chimeras using standard procedures.

The retroviral OmniBank Vector VICTR24 (S1A Fig) contained a splice acceptor sequence (SA) followed by a 5’ selectable marker β-GEO, a functional fusion between the β-galactosidase and neomycin resistance genes, for identification of successful gene trap events followed by a polyadenylation signal (pA). Insertion of the retroviral vector into STX1B led to the splicing of the endogenous upstream exons into this cassette to produce a fusion transcript that was used to generate a sequence tag (OST) of the trapped gene by 3′ RACE [82]. More information on the gene trap strategies can be obtained from the TIGM website (http://www.tigm.org/). Chimeric mice were born three weeks later. Male chimeras where then mated with wt C57BL/6 to obtain germline transmission. We obtained four founders and used them to establish the colony. The derived F1 mice were screened by PCR. Genotyping of tail DNA was accomplished using PCR with forward and backward primers for the wt locus (5’-AAT CCG AAC AGA CTG AGA TAC ATT -3’; 5’-aGA GTT GGG CGG AAG GTA CAA GAG -3’) and two primers for the LTR mutant locus (5’-ATA AAC CCT CTT GCA GTT GCA TC-3’; 5’-AAA TGG CGT TAC TTA AGC TAG CTT GC-3’). A 330-bp band was amplified for homozygous wt mice, a 270-bp and 200-bp band for homozygous mutant mice, and the three bands for heterozygous mice (S1A and S1B Fig). Western blot analyses demonstrated absence of STX1B protein in STX1B mutants (S1C Fig).

STX1A mutant mice were a kind gift from Thomas Sudhof [83]. STX1A and STX1B mutant mice were mated to produce double heterozygous mutant mice. The double heterozygous mutant mice were then mated with each other, and the genotypes of their offspring were determined by PCR.

Open-book preparations of mouse spinal cords were essentially done as described above for chicken embryos. Embryos were collected and dissected at E12. Spinal cords were removed, opened at the roof plate and pinned down in a Sylgard dish in 4% PFA for 20–50 minutes. Commissural axons were traced with Fast DiI (5 mg/ml in ethanol) by incubating the open-book preparations in PBS for at least 3 days before mounting in PBS between two 24x24 mm coverslips sealed with vacuum grease.

### Ethics statement

Animal experimentation was conducted according to the European and National (Spanish) guidelines. The experimental protocol was approved by the local University Committee (CEEA-UB, Comitè Ètic d´Experimentació Animal de la Universitat de Barcelona) and by the Catalan Government (Generalitat de Catalunya, Departament de Territori I Sostenibilitat) with the approval number #9431.

### Immunoblots

Embryonic brains were lysed in hypotonic buffer (150 mM NaCl, 50 mM Tris pH 7.2, 2 mM EDTA, 1% Triton X-100, and protease inhibitors (Complete, Mini Protease Inhibitor Cocktail Tablets, Roche)).

SDS-sample buffer was added to the lysates, and the proteins were analyzed by SDS-PAGE and Western blot. Proteins were transferred onto nitrocellulose membranes, which were blocked with 5% non-fat dry milk in Tris-HCl buffered saline (TBS) containing 0.1% Tween 20, and incubated overnight at 4°C with mouse anti-STX1 (HPC-1 clone 1:500–1000, Sigma) and anti-actin (1:10000, Millipore) antibodies. After incubation with secondary antibodies, blots were developed following the ECL method (Amersham Pharmacia Biotech).

### Dissociated neuronal cultures

Spinal cord dorsal neurons were isolated from E11 mouse embryos. After dissection of the alar plate, tissue was treated with Trypsin 1x for 3 minutes at 37°C and with DNase and Fetal Bovine Serum at 37°C for 10 minutes. 50000 cells were seeded on 200 mm^2^ wells, previously treated with 0.5 mg/mL poly-D-lysin coverslips. Cells were maintained in vitro for 16–24 hours in Neurobasal medium with B27 1x, Glutamax 1x, Penicillin/Streptomycin 1x and Fetal Bovine Serum 10%. Neurons were fixed in 4% paraformaldehyde, and incubated with mouse anti-DCC (BD Pharmigen) and goat anti-Robo3 (RD Systems) antibodies (1:100). Mouse anti-STX1 (Sigma), mouse anti-VAMP2 (Synaptic Systems), mouse anti-SNAP25 (Covance); mouse anti-Ti-VAMP (Abcam) were incubated in blocking buffer to a final concentration of 1:100. Double immunodetection for DCC and SNARE proteins required incubation in blocking buffer containing anti-mouse IgG Fab specific antibodies. Alexa 488 anti-mouse and anti-goat secondary antibodies, and Alexa 568 anti-goat antibodies were incubated in blocking buffer (1:100). Neurons were imaged in a Leica TCS SP5 confocal microscope using a 63x oil-immersion objective.

To analyse Robo3 receptor surface expression, dorsal spinal cord neurons from wild-type and STX1A/B KO embryos were prepared as above. We followed the protocol described in [15]. Briefly, cultures were incubated with either 300 ng/ml of Netrin-1 (+Netrin-1) or with BSA (-Netrin-1) for 30 min at 37°C. After fixation, cultures were blocked with 10% of Normal Horse Serum (NHS) in PBS for 2 hours and incubated without detergent with primary goat anti-Robo3 (RD Systems, Robo3 1:100). Afterwards, cells were washed with PBS and incubated with secondary antibodies (Alexa Fluor 488 anti-goat, 1:50) in excess to block all the primary antibody epitopes. After several washes in PBS, cells were blocked again with 10% NGS and incubated with goat anti-Robo3 primary antibody, in the presence of 0,3% Triton X-100. Cells were then washed and incubated with Alexa Fluor 568 anti-goat (both at 1:50, Jackson). Cells were washed, stained with DAPI and mounted in Mowiol. Cells were imaged in a Leica TCS SP5 microscope using a 63x oil-immersion objective. Z stacks of 8–12 confocal planes were acquired and images were processed with Fiji software. 20 growth cones per condition were analyzed with GraphPad software to quantify surface and inside receptor expression. Statistics were calculated with a two-way ANOVA and a Tukey’s multiple comparison post-test, p<0.05.

### Explant cultures

Dorsal (alar plate) spinal cord explants were dissected from E11 mouse embryos. Tissue explants were co-cultured as described [16], along with cell aggregates of HEK293T cells stably transfected with a pCEP4-rNetrin-1c-myc construct or with control HEK293T cells. Explants and cell aggregates were embedded in a collagen matrix and maintained in vitro for 48h in Neurobasal supplemented media (Penecillin/Streptomycin 1x, Glutamax 1x and B27 1x). Cultures were fixed with 4% paraformaldehyde and immunolabeled with mouse anti-βIII-tubulin (1:1000, Covance) in blocking buffer. Images were acquired with an Eclipse E1000 microscope using a 10x objective. Axon elongation was quantified by calculating the area occupied by the axons in both the proximal and distal quadrants. Data was statistically analysed as above.

Explants of commissural neurons were dissected from untreated and experimental chicken embryos at HH25. Explants were cultured in serum-free DMEM medium with GlutaMax, 5 mg/ml Albumax, N3 and 1 mM sodium pyruvate. Eight-well Lab-Tek dishes (Nunc) were coated with polylysine (20 μg/ml) and laminin (10 μg/ml). After 24 h control medium or medium containing Slit-2 (100 ng/ml; R&D Systems) was added to the explants. After an additional 18 h, cultures were fixed in 4% PFA and the average neurite length of each explant was measured using the cellSens program (Olympus).

## Supporting information

S1 Fig Diagram of the generation of STX1B mutant mice. (A) Illustration of mutation in the STX1B is shown. The insertion is in the first intron of the gene. (B) Agarose gel illustrating genotyping bands for wt, heterozygous, and STX1B KO mice. (C) Western blot showing the absence of Syntaxin 1B protein in STX1B mutant mice but not in wt or heterozygous mice. Actin protein was used as loading control.
(PDF)

S2 Fig STX1A/B double mutant embryos show aberrant Robo3-immunolabeled commissural axons. E12 mouse spinal cords were immunostained with α-Robo3 antibodies to detect commissural axons; sections were counterstained with DAPI. Low magnification views (A,C) and enlarged views (B,D) are shown. In wt embryos Robo3-stained fibers form a tight bundle of axons directed towards the floor plate (A). In STX1A/B KO embryos Robo3-stained commissural fibers invade lateral domains of the ventral spinal cord (red arrowheads in C). In addition, many ipsilateral commissural fibers are tipped with growth cones in STX1A/B mutants (arrowheads in D). Consistently, the number of Robo3-stained contralateral fibers is decreased in STX1A/B knock-out embryos when compared to control embryos (arrows in A,C). Scale bar: 100μm (A), 50μm (B).
(TIF)

S3 Fig Robo3 surface expression in STX1A/B knock-out commissural neurons. Commissural neurons from E11 wild-type (A, B) and STX1A/B null mutant embryos were cultured (C, D) and incubated with recombinant Netrin-1 (B,D) or with control medium (A,C). Cultures were immunostained for the labeling of surface Robo3 receptor (green color) and the intracellular pool of Robo3 protein (red color). Analyses of the images suggested no major changes in wild-type growth cones incubated with Netrin-1 (A,B) and a slight increase in the intracellular Robo3 signal in STX1A/B knock-out growth cones treated with Netrin-1 (C,D). Quantification of outside/inside signals confirmed that incubation with Netrin-1 results in a significant increase (two-way ANOVA; p<0.05 **p≤0,05) of Robo3 intracellular signal in STX1A/B deficient growth cones (E). Scale bar: 5μm.
(TIF)
