# Supplementary figures and images for: A conserved role for Syntaxin-1 in pre- and post-commissural midline axonal guidance in fly, chick, and mouse
Source: PLoS Genet. 2018 Jun 18;14(6):e1007432. doi: 10.1371/journal.pgen.1007432 (PMC6029812; doi:10.1371/journal.pgen.1007432)

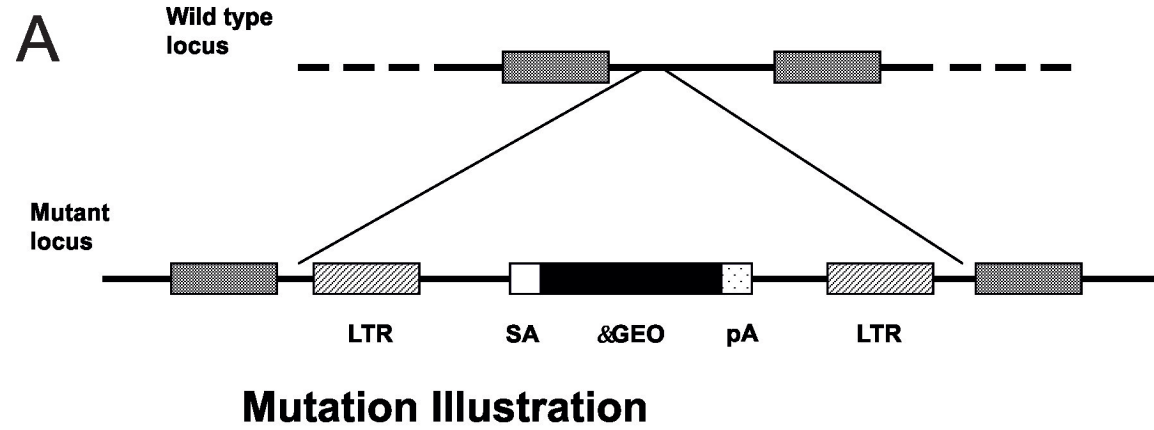

Accession: NM\_024414

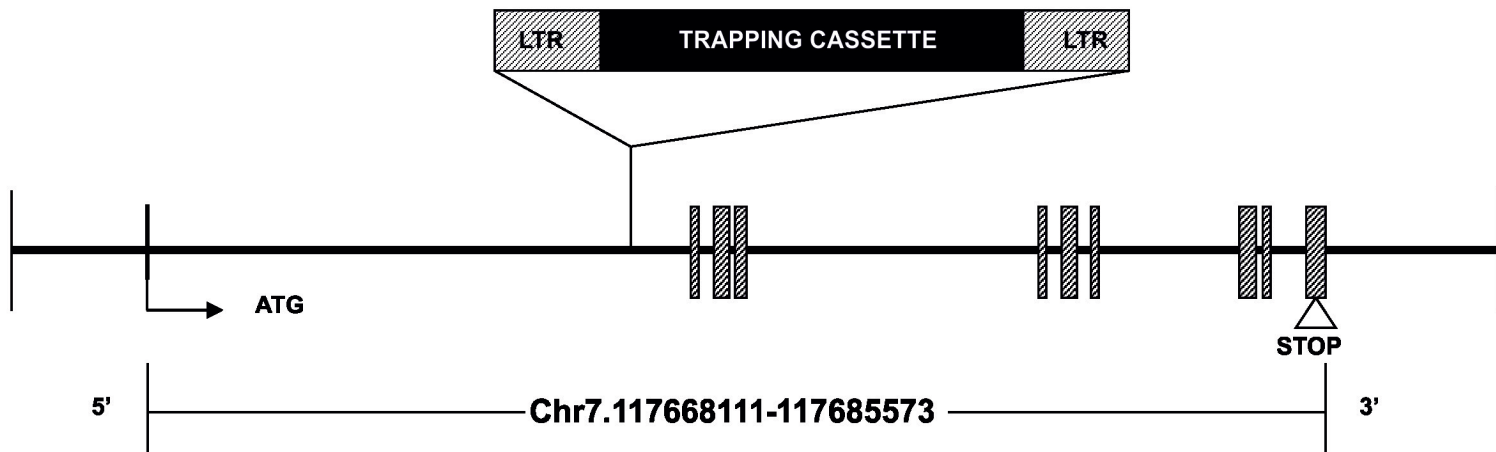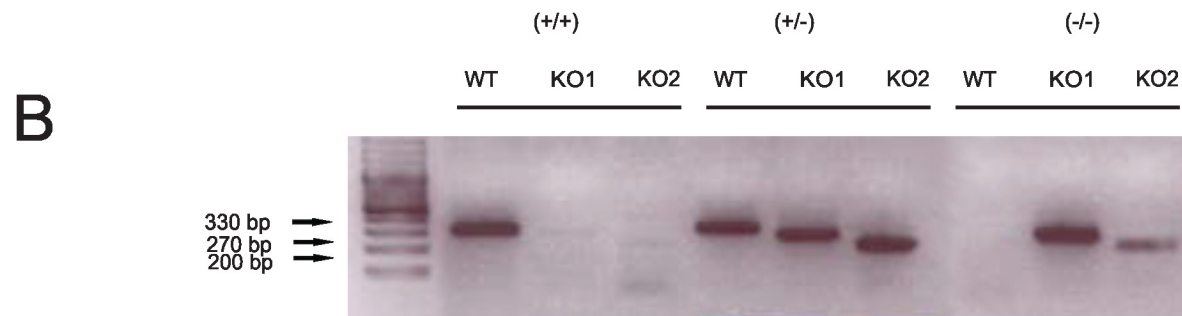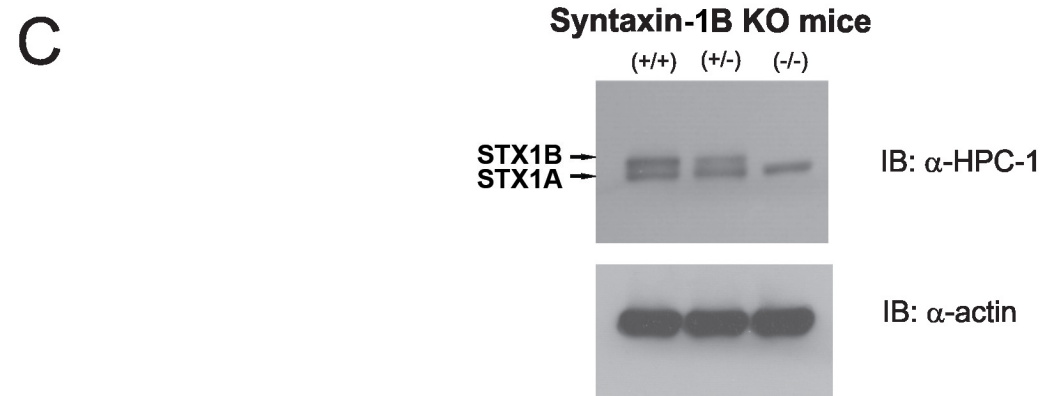

Supplement: S1 Fig — (A) Illustration of mutation in the STX1B is shown. The insertion is in the first intron of the gene. (B) Agarose gel illustrating genotyping bands for wt, heterozygous, and STX1B KO mice. (C) Western blot showing the absence of Syntaxin 1B protein in STX1B mutant mice but not in wt or heterozygous mice. Actin protein was used as loading control. (PDF) [file pgen.1007432.s001.pdf]

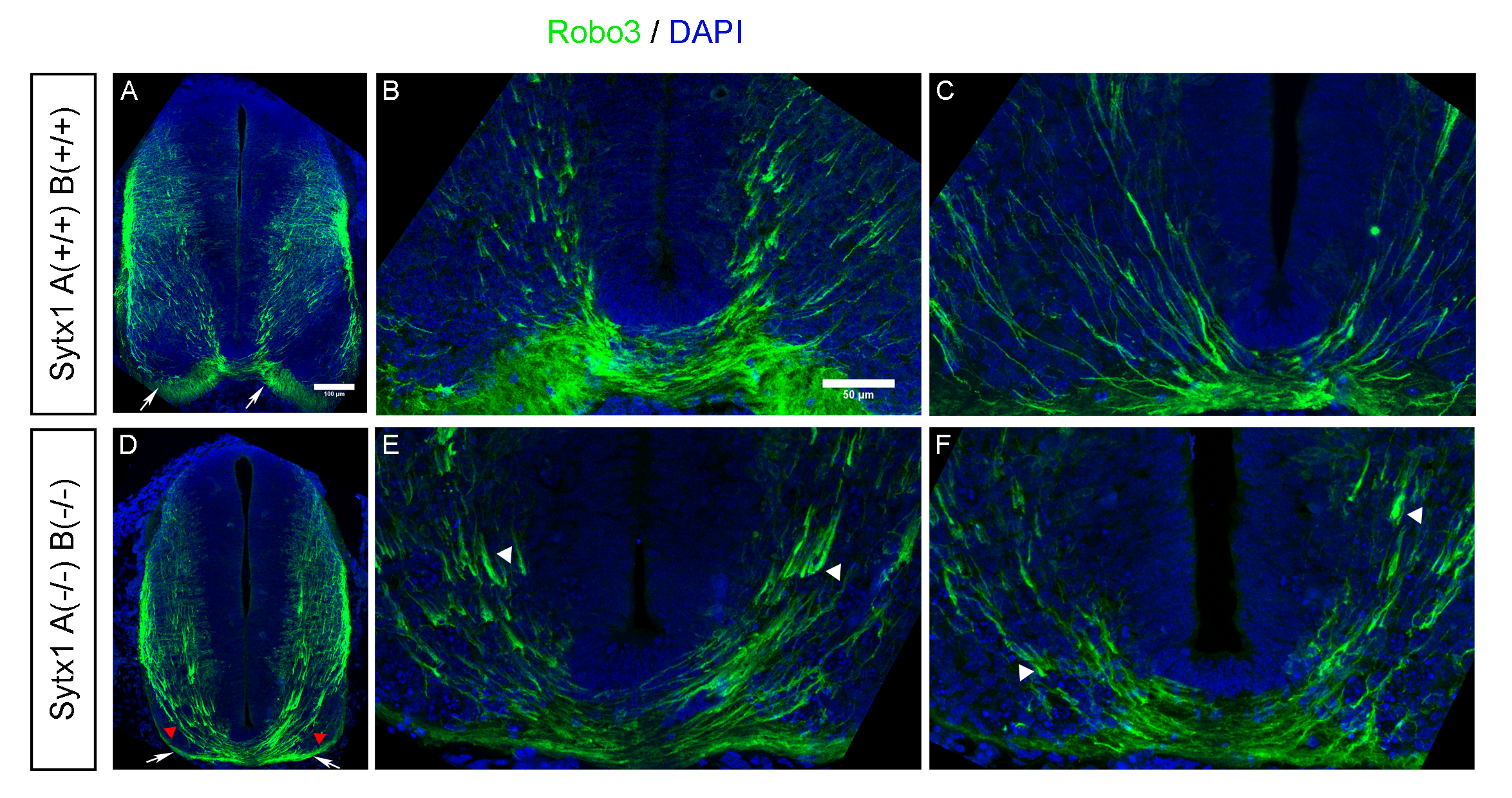

Supplement: S2 Fig — E12 mouse spinal cords were immunostained with α-Robo3 antibodies to detect commissural axons; sections were counterstained with DAPI. Low magnification views (A,C) and enlarged views (B,D) are shown. In wt embryos Robo3-stained fibers form a tight bundle of axons directed towards the floor plate (A). In STX1A/B KO embryos Robo3-stained commissural fibers invade lateral domains of the ventral spinal cord (red arrowheads in C). In addition, many ipsilateral commissural fibers are tipped with growth cones in STX1A/B mutants (arrowheads in D). Consistently, the number of Robo3-stained contralateral fibers is decreased in STX1A/B knock-out embryos when compared to control embryos (arrows in A,C). Scale bar: 100μm (A), 50μm (B). (TIF) [file pgen.1007432.s002.tif]

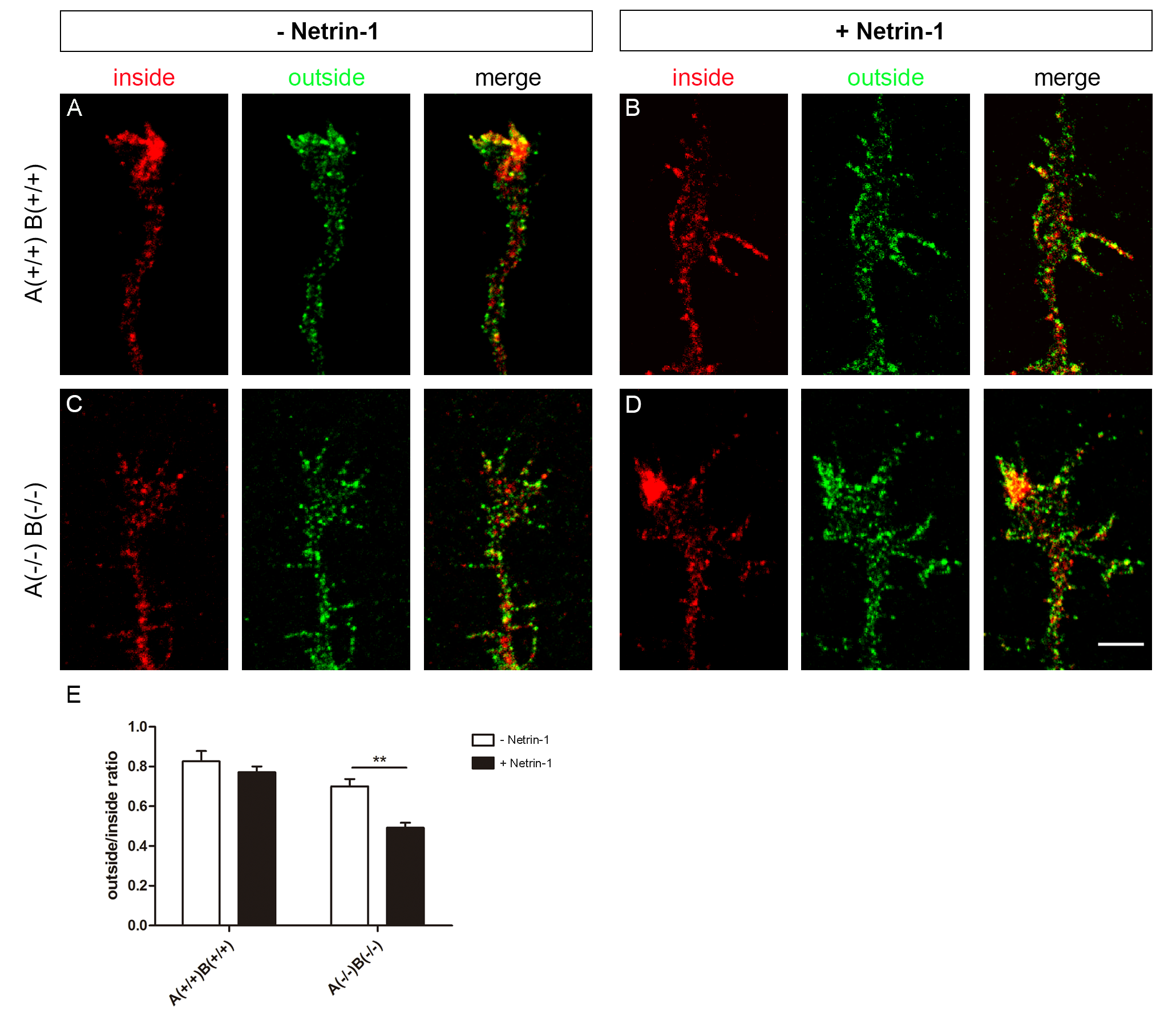

Supplement: S3 Fig — Commissural neurons from E11 wild-type (A, B) and STX1A/B null mutant embryos were cultured (C, D) and incubated with recombinant Netrin-1 (B,D) or with control medium (A,C). Cultures were immunostained for the labeling of surface Robo3 receptor (green color) and the intracellular pool of Robo3 protein (red color). Analyses of the images suggested no major changes in wild-type growth cones incubated with Netrin-1 (A,B) and a slight increase in the intracellular Robo3 signal in STX1A/B knock-out growth cones treated with Netrin-1 (C,D). Quantification of outside/inside signals confirmed that incubation with Netrin-1 results in a significant increase (two-way ANOVA; p<0.05 **p≤0,05) of Robo3 intracellular signal in STX1A/B deficient growth cones (E). Scale bar: 5μm. (TIF) [file pgen.1007432.s003.tif]
